# Haplotype-Resolved Genome Analyses Reveal Genetically Distinct Nuclei within a Commercial Cultivar of *Lentinula edodes*

**DOI:** 10.3390/jof8020167

**Published:** 2022-02-09

**Authors:** Qi Gao, Dong Yan, Shuang Song, Yangyang Fan, Shouxian Wang, Yu Liu, Yu Huang, Chengbo Rong, Yuan Guo, Shuang Zhao, Wentao Qin, Jianping Xu

**Affiliations:** 1 Beijing Engineering Research Center for Edible Mushroom, Institute of Plant Protection, Beijing Academy of Agriculture and Forestry Sciences, 9 Shuguang Garden Zhonglu, Haidian District, Beijing 100097, China; gaoqi@hotmail.co.jp (Q.G.); songshuang@ipepbaafs.cn (S.S.); fanyy@pku.edu.cn (Y.F.); wangshouxian@ipepbaafs.cn (S.W.); liuyu@ipepbaafs.cn (Y.L.); huangyu@ipepbaafs.cn (Y.H.); rongchengbo@ipepbaafs.cn (C.R.); guoyuan@ipepbaafs.cn (Y.G.); zhaoshuang@ipepbaafs.cn (S.Z.); qinwentao@ipepbaafs.cn (W.Q.); 2 State Key Laboratory of Protein and Plant Gene Research, Peking-Tsinghua Center for Life Sciences, School of Advanced Agricultural Sciences, Peking University, Beijing 100871, China; 3 College of Agriculture and Food Engineering, Baise University, 21 Zhongshan Second Street, Youjiang District, Baise 533000, China; 4 Institute of Agri-food Processing and Nutrition, Beijing Academy of Agricultural and Forestry Sciences, Beijing 100097, China; 5 Department of Biology, McMaster University, Hamilton, ON L8S 4K1, Canada

**Keywords:** *Lentinula edodes*, de novo, comparative genomics, hybridization, dikaryon, dedikaryotization, monokaryons, gene enrichment, mating-type loci

## Abstract

*Lentinula edodes* is a tetrapolar basidiomycete with two haploid nuclei in each cell during most of their life cycle. Understanding the two haploid nuclei genome structures and their interactions on growth and fruiting body development has significant practical implications, especially for commercial cultivars. In this study, we isolated and assembled the two haploid genomes from a commercial strain of *L. edodes* using Illumina, HiFi, and Hi-C technologies. The total genome lengths were 50.93 Mb and 49.80 Mb for the two monokaryons SP3 and SP30, respectively, with each assembled into 10 chromosomes with 99.63% and 98.91% anchoring rates, respectively, for contigs more than 100 Kb. Genome comparisons suggest that two haploid nuclei likely derived from distinct genetic ancestries, with ~30% of their genomes being unique or non-syntenic. Consistent with a tetrapolar mating system, the two mating-type loci A (matA) and B (matB) of *L. edodes* were found located on two different chromosomes. However, we identified a new but incomplete homeodomain (HD) sublocus at ~2.8 Mb from matA in both monokaryons. Our study provides a solid foundation for investigating the relationships among cultivars and between cultivars and wild strains and for studying how two genetically divergent nuclei coordinate to regulate fruiting body formation in *L. edodes*.

## 1. Introduction

*Lentinula edodes*, commonly known as Xianggu in Chinese, is one of the most popular cultivated mushrooms, especially in East Asia [1,2]. Globally, *L. edodes* is the second most widely cultivated mushroom species, with the production of *L. edodes* in 2019 reaching 11 million tons in China alone. Aside from its delicious taste, *L. edodes* is also rich in multiple nutrients and medicinal properties, such as immunity-strengthening, antitumor, antioxidant, anti-hypertensive, hypocholesterolemic, and antithrombotic activities [3]. 

East Asia is known as the center of *L. edodes* germplasm diversity and the geographic origin of domestication for this mushroom [4]. It is estimated that there are about 500 cultivars distributed throughout China for commercial cultivation. However, the genetic relationships among these cultivars and how these cultivars are related to wild strains remain largely unknown [5]. Knowledge about such relationships can provide crucial information for future breeding experiments. In addition, despite the agricultural, economic, and medicinal importance of *L. edodes*, the genome characteristics of this mushroom remain poorly known [6]. High-quality reference genomes can serve as crucial resources for mushroom breeding, population genetics, and comparative genomic studies [7]. While several studies have published genome sequences of strains of *L. edodes* [8,9], due to limitations of the applied technology, those genome assemblies were generally incomplete, containing hundreds or more contigs, with little or no chromosomal level information. A high-quality chromosome-level reference genome has significant advantages for studying the biology and evolution of edible cultivated mushroom species [7]. At present, only one haploid genome from a dikaryotic cultivar L808 is reported to be assembled to the chromosome level (BioSample: SAMN14591202, https://www.ncbi.nlm.nih.gov/biosample/SAMN14591202, accessed on 22 December 2021). However, details about the specific components of this genome, such as mating-type loci, of this monokaryon are still lacking. Hi-C (high throughput chromosome conformation capture) technology can identify the interactions between points in the whole genome to provide high-quality chromosome-level assemblies [10]. This technology has been widely used in plant genome assembly to not only analyze the three-dimensional spatial structure of each chromosome but also help provide haplotype-resolved assemblies within individual genomes [11]. In this study, we use the Hi-C technology to provide high-quality chromosome level assemblies for two haploid nuclei within a well-known cultivar of *L. edodes*.

From a life cycle perspective, *L. edodes* is a tetrapolar basidiomycete, and most of its young dikaryotic hyphal cells contain two haploid nuclei each and share a single cytoplasm without undergoing nuclear fusion [12]. Only dikaryon can form primordia and develop into fruiting bodies in this and many other mushroom species. How the two nuclei interact with each other within each cell and how the chromosomal structural differences between these two nuclei could impact meiosis and sexual spore viability are largely unknown in this species. Genome recombination during meiosis is essential for generating genetically diverse progeny of *L. edodes* and other basidiomycetes for breeding purposes. Understanding the genome structure and gene content differences between the two haploid nuclear genomes would represent a significant step towards understanding the roles of the two nuclei in the life cycle of *L. edodes*. In addition, combined with population genetics methods, phenotypic traits can be more accurately associated with different nuclei of a parental dikaryon. Such knowledge not only can improve the accuracy of genetic mapping but can also help establish reliable markers for breeding phenotypically superior strains. Recently, high-fidelity (HiFi) sequence data from PacBio in CCS (circular consensus sequencing) have provided impressive results on assembly and variant detection, showing its potential in resolving complex genome regions [13]. 

In this study, using the Illumina, HiFi, and Hi-C technologies, we assembled the genomes of the two nuclei within a widely cultivated dikaryotic *L. edodes* strain. Our analyses revealed significant genomic differences between the two monokaryons, including chromosomal rearrangements and a large number of nucleus-specific genes. We discuss the implications of these results for strain relationship tracking and understanding the interactions between them during growth, secondary metabolite production, heterokaryon formation, and fruiting body development in *L. edodes*.

## 2. Materials and Methods

### 2.1. L. edodes Strains and DNA Preparation

The dikaryotic *L. edodes* strain JZB2102217 is a widely grown commercial cultivar in northern China. The strain is maintained at the Beijing Germplasm Resource Bank for Edible Fungi. We used lywallzyme (Guangdong Institute of Microbiology, Guangzhou, China) to digest the cell walls of the diploid cultivar JZB2102217 and obtained protoplasts. The protoplasts were diluted and revived on a cell wall regeneration medium to form hyphae and colonies. Hyphae without clamp connections were identified as putative monokaryons [14]. Subsequently, we mated these putative monokaryons with each other to select monokaryons with compatible mating types. Sexually compatible monokaryons will mate and form dikaryon with clamp connections. Through this selection protocol, we obtained two monokaryotic strains, SP3 and SP30. These two monokaryotic strains, SP3 and SP30, were crossed with each other to form a re-constituted dikaryon 3 × 30.

For high-quality DNA extraction from strains SP3 and SP30, vegetative mycelia of SP3 and SP30 were grown in a liquid MG medium (2% malt extract, 2% glucose) for 15 days at 25 °C in darkness. The mycelia were collected by centrifugation, washed with 0.1 M PBS, and frozen in liquid nitrogen. High-quality genomic DNA was extracted from the mycelia using the CTAB method [15]. RNase A (Leagene, Beijing) in 10 µg/mL was used to remove RNA from the samples. The quality and quantity of the extracted DNA were examined using a NanoDrop 2000 spectrophotometer (NanoDrop Technologies, Wilmington, DE, USA), Qubit dsDNA HS Assay Kit on a Qubit 3.0 Fluorometer (Life Technologies, Carlsbad, CA, USA), and electrophoresis on a 0.8% agarose gel, respectively.

### 2.2. Genome Sequencing and Assembly

The extracted DNA molecules were sequenced with both the Illumina HiSeq X Ten (Illumina Inc., San Diego, CA, USA) at the Beijing Novogene Bioinformatics Technology Co., Ltd., and the PacBio Sequel II (Pacific Biosciences of California, Menlo Park, CA, USA) platform at the Frasergen Bioinformatics Co., Ltd. (Wuhan, China). The short reads from the Illumina platform were quality-filtered by high-throughput quality control (HTQC) [16] using the following method. First, we removed reads that contained low-quality bases (quality value ≤20) exceeding 40%, as well as the reads with ambiguous base calls for over 10% of each read. Next, we removed reads whose overlap with adapter exceeded 15 bp. The quality-filtered reads were then used for genome size estimation. We preliminary assembled with SOAP de novo software and adjusted parameters to select the optimal k-mer and the least scaffolds [17]. We generated the 15-mer occurrence distribution of sequencing reads from short libraries for both strains SP3 and SP30.

SMRTbell libraries were sequenced on a PacBio Sequel II system, and consensus reads (HiFi reads) were generated using ccs software (https://github.com/pacificbiosci-ences/unanimity, accessed on 22 December 2021) with the parameter “--inPasses 3”. We generated 8.65 Gb and 5.35 Gb PacBio HiFi reads of SP3 and SP30, respectively. These long (~15 kb) and highly accurate (>99%) HiFi reads were assembled using HiCanu [18] for both strains with default parameters.

The vegetative mycelia of SP3 and SP30 were also used to generate Hi-C DNA libraries at the Frasergen Enterprise (Wuhan, China). Hi-C libraries were constructed following protocols described in a previous study [19] with minor modification. Briefly, fungal samples were ground into powder in liquid nitrogen, and then incubated with lysozyme at 37 °C for 10 min to digest the cell wall. Then cells were cross-linked for 10 min with 3% formaldehyde at room temperature and quenched with 0.37 M final concentration glycine for 5 min. The cross-linked cells were subsequently lysed. Endogenous nucleases were inactivated with 0.3% SDS. The chromatin DNA was digested by 100U MboI (NEB), labeled with biotin-14-dCTP (Invitrogen, Carlsbad, CA, USA), and then ligated by 50U T4 DNA ligase (NEB). After reversing cross-links, the ligated DNA was extracted and purified through a QIAamp DNA Mini Kit (Qiagen, Hilden, Germany), according to the manufacturer’s instructions. Purified DNA was sheared into fragments of 300–500 bp, followed by blunt-end, repair, additions of an A-tail and adaptor-added, purification through biotin-streptavidin-mediated pull-down, and PCR amplification. Finally, the Hi-C libraries were quantified and sequenced on the Illumina Nova-seq platform (San Diego, CA, USA). The chimeric fragments from the original cross-linked long-distance physical interactions were then isolated and processed into libraries. For anchored contigs, 34,909,404 and 31,267,524 clean reads were generated from the Hi-C library and were mapped to the SP3 and SP30 preliminary assembly using Juicer [20] with default parameters. Paired reads mapped to different contigs were used for the Hi-C associated scaffolding. Self-ligated, non-ligated, and other invalid reads were filtered out. We applied 3D-DNA to order and orient the clustered contigs. Then, Juicer was used to filter the sequences and cluster them, and the Juicebox was applied to adjust chromosome construction manually (Figure S1). We finally anchored the scaffolds on ten chromosomes. In addition, the BUSCO v3.0.231 pipeline was used to assess the completeness and accuracy of the SP3 and SP30 genomes.

### 2.3. Genome Annotation

Two methods, homology-based and de novo prediction, were combined to identify the repeat contents in SP3 and SP30 genomes. We identified known transposable elements (TEs) within the genomes using RepeatMasker [21] with the Repbase TE library [22,23]. RepeatProteinMask searches were also conducted using the TE protein database as a query library. We constructed a de novo repeat library using RepeatModeler (open-1.0.11) (http://www.repeatmasker.org/RepeatModeler/, accessed on 28 July 2021), which automatically executed two core de novo repeat finding programs, RECON [24] and RepeatScout [25], to comprehensively conduct, refine, and classify consensus models of putative interspersed repeats. Furthermore, we performed a de novo search for long-terminal repeat (LTR) retrotransposons against the SP3 and SP30 genome sequences using LTR_FINDER [26] and identified tandem repeats using the Tandem Repeat Finder (TRF) package [27].

We predicted protein-coding genes of the SP3 and SP30 genomes using ab initio gene prediction, homology-based gene prediction, and RNA-seq aided gene prediction. Prior to gene prediction, the assembled SP3 and SP30 genomes were hard and soft masked using RepeatMasker. We adopted Augustus [28,29,30] and Genescan [31] to perform ab initio gene prediction. Models used for each gene predictor were trained from a set of high-quality proteins generated from the RNA-seq dataset. We used Exonerate [32] to conduct homology-based gene prediction. First, the protein sequences were aligned to our genome assembly and predicted protein-coding genes using Exonerate with the default parameters. To carry out the RNA-seq-assisted gene prediction, we first assembled RNA-seq clean reads into transcripts using TopHat [33], and the gene structures were formed using Cufflinks [34]. Finally, Maker [35] was used to integrate the prediction results of the three methods to predict gene models. The output included a set of consistent and non-overlapping sequence assemblies, which were used to describe the gene structures. 

Gene functions were inferred according to the best match of the alignments to the National Center for Biotechnology Information (NCBI), Non Redundant (NR), TrEMBL [36], InterPro [37], and Swiss Prot [36] protein databases using BLASTP [38,39] and the Kyoto Encyclopedia of Genes and Genomes (KEGG) database [40] with an E value threshold of 1 × 10^−5^. The protein domains were annotated using InterProScan [41] based on InterPro protein databases. The Gene Ontology [42] (GO) ID for each gene was obtained from Blast2GO [43]. Carbohydrate-active enzymes (CAZymes) were analyzed using the CAZyme database (http://www.cazy.org/, accessed on 15 November 2021).

We used tRNAscan SE algorithms [44] with default parameters to identify the genes associated with tRNA, which is an adaptor molecule composed of RNA used in biology to bridge the triplet code in mRNA with the twenty letter code of amino acids in proteins. For ribosomal RNA (rRNA) identification, we first downloaded rRNA sequences in closely related species from the Ensembl database. Then rRNAs in the database were aligned against our genomes using blastn [38,39] with a cutoff E value < 1 × 10^−5^, a sequence identity ≥85%, and a match length ≥50 bp. MiRNAs and snRNAs were identified by Infernal [45] software against the Rfam database [46] with default parameters.

### 2.4. Comparative Genomic Analysis

The all versus all blastp method (E value < 1 × 10^−5^) was used to detect orthologous genes between the SP3 and SP30 genomes. SNPs and indels were identified based on genomic alignment results between shared genes in the SP3 and SP30 genomes using the MUMmer [47] and LASTZ [48,49] tools. Then, syntenic paralogous blocks were identified with MCSCAN [50] between our genomes and the deposited genomes of *L. edodes* strains L808, NBRC111202, B17, and W1-26, which were all monokaryotic isolates with complete gene annotation available on NCBI or GGI. According to the results of collinearity analysis, the genes that all *L. edodes* strains shared in the same block and the same order were recognized as the synteny genes of *L. edodes*. Shared genes but without the collinear relationship between SP3 and SP30 were considered rearranged genes between SP3 and SP30. The gene sequences of the rearranged genes between SP3 and SP30 were extracted from the SP3 and SP30 genome sequences and compared with each other through blastn (E value < 1 × 10^−5^). Genes without any aligned partners between the two strains were considered unique genes of strains SP3 or SP30. We randomly selected several unique genes to design primers for PCR amplification of 500–800 bp fragments for confirmation of their unique presence in strains SP3 and SP30. Each reaction was performed in 20 μL of total volume: 1 μL of each primer (10 μM), 2 μL of DNA, 10 μL of PrimeSTAR^®^ Max DNA Polymerase (TAKARA), and 6 μL of ddH2O. The PCR was performed as follows: 95 °C, 5 min; 30 cycles of 30 s at 95 °C, 30 s at 55 °C, and 40 s at 72 °C; 72 °C, 10 min. The PCR products were checked through electrophoresis on 0.8% agarose gel.

### 2.5. Identification of Mat-Genes

The matA genes were identified by mapping the annotated genome and proteome sequences to the mating-type associated proteins of the mitochondrial intermediate peptidase (*mip*) genes in *Coprinopsis cinerea* and *Schizophyllum commune*. For matB genes, the pheromone receptor genes in SP3 and SP30 were identified by the Swissprot annotation with the keyword “Pheromone receptor”. The sequence length of pheromone precursor is very short (usually 50–60 amino acids), and thus they could not be predicted in the normal genome annotation procedure. Instead, the Expasy (https://web.expasy.org/translate/, accessed on 19 October 2021) software with Pfam search was used to annotate pheromone precursor by searching the ~20 kb flanking sequence of pheromone receptor genes.

### 2.6. Data Availability and Image Analysis 

All datasets reported in this study have been deposited in the Genome Warehouse in National Genomics Data Center, Beijing Institute of Genomics, Chinese Academy of Sciences/China National Center for Bioinformation, under BioProject ID PRJCA007678 (https://ngdc.cncb.ac.cn/bioproject/browse/PRJCA007678, accessed on 22 December 2021). The *L. edodes* SP3 and SP30 genomes and annotations are publicly accessible under accession numbers GWHBGWX00000000 and GWHBGWY00000000, respectively. In this article, genome circos images, collinearity images, etc., were drawn using the TBtools software. KEGG and GO enrichment analyses were performed using the OmicShare tools (www.omicshare.com/tools, accessed on 22 December 2021). Significantly enriched pathways and GO terms among rearranged genes were compared to syntenic genomes by the hypergeometric test. The calculated *p*-value was adjusted through FDR correction, taking FDR ≤ 0.05 as a threshold. Pathways and GO terms meeting this condition were defined as significantly enriched. CAZyme cluster analysis images were drawn with excel. The mating-type loci structure diagrams were drawn using the snapgene software combined with photoshop. 

To generate the Z-stack image, we selected a mature fruiting body of strain 3 × 30 and removed the gills piece by piece with a tweezer. The gill pieces were fixed in 4% para-formaldehyde for 1 h at 4 °C. We used the optimized cryosections technology (patent protection, application number: 202010861707.7) to section the gills with a thickness of 1 μm. Then we dyed the sections with 8 µg/mL DAPI (Sigma–Aldrich, St. Louis, MO, USA) and used a confocal microscope (LSM 900 with Airyscan2, Zeiss, Germany) to observe the chromosome structure after cell fusion in the basidia under 100× oil lens. We took the picture with Z-stacked photography. We used ImageJ software with the Fiji plugin for 3D analysis, following the method reported by Sawithree et al. [51].

## 3. Results

### 3.1. Genome Sequencing, Assembly, and Annotation

**Figure 1:**
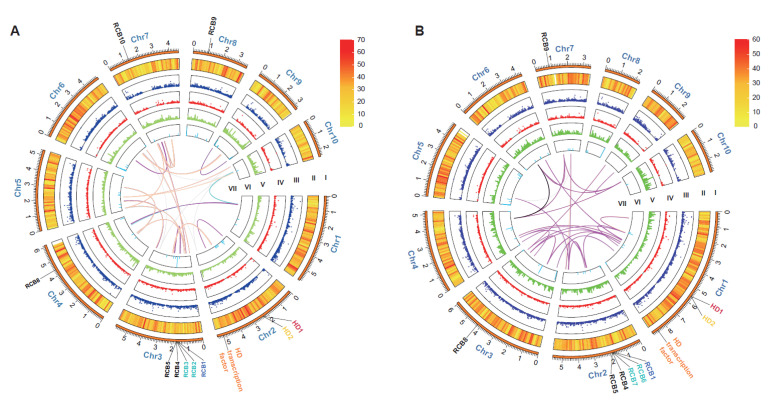
Global view of two haploid genomes within a dikaryotic cultivar of *Lentinula edodes*. (**A**) the SP3 genome. (**B**) the SP30 genome. The gene names marked outside the circle were annotated as mating-type-related genes; homeodomain (HD)**,** pheromone receptor (RCB). Circle I represents the 10 pseudo-chromosomes of *L. edodes* (Mb). Circle II represents annotated gene density on each pseudo-chromosome in 100-kb non-overlapping windows. Circle III represents SNP positions of alleles on two the monokaryons, and each point signifies one SNP. Circle IV represents indel positions of alleles on the two monokaryons, and each point means one Indel. Circle V represents the density of repetitive sequences on each pseudo-chromosome in 10-kb non-overlapping windows. Circle VI represents rRNA, snRNA, and tRNA densities on each pseudo-chromosome in 10-kb non-overlapping windows. Circle VII represents intra genomic collinearity. Peachpuff lines mean collinearity genes have been annotated by both InterPro and KEGG; Magenta lines mean collinearity genes have been annotated by InterPro; Cyan lines mean collinearity genes have been annotated by KEGG; Grey lines means hypothetical proteins.

The genomes of monokaryons SP3 and SP30 of *L. edodes* cultivar JZB2102217 were sequenced using Illumina HiSeq X Ten and PacBio Sequel II platforms. Approximately 8.65 Gb and 5.35 Gb PacBio HiFi reads of SP3 and SP30 were assembled using HiCanu [18]). We obtained preliminary assemblies of the SP3 and SP30 genomes into approximately 362 and 283 contigs with a total size of 59.33 Mb and 56.44 Mb, and 4.76 Mb and 2.53 Mb of N50, respectively. High-throughput chromatin conformation capture (Hi-C) libraries were constructed to scaffold the contig-assembly. Further, 34,909,404 and 31,267,524 clean reads were mapped to the polished SP3 and SP30 genome and applied to order and orient the clustered contigs by Lachesis [52]. Using the interaction mapping for correction, we assembled a total length of 50.83 Mb of the SP3 genome with 10 chromosomes (including 18 contigs), contigs N50 = 3.40 Mb, scaffold N50 = 5.32 Mb, with a chromosome anchoring rate of 99.63% for contigs longer than 100 Kb. For the SP30 genome, the total length was 49.80 Mb with 10 chromosomes (including 18 contigs), contigs N50 = 2.64 Mb, scaffold N50 = 5.24 Mb, with a chromosome anchoring rate of 98.91% for contigs longer than 100 Kb (Table 1 and S1; Figure 1). According to interaction maps for the SP3 and SP30 genomes, Hi-C-assisted assembly of both genomes confirmed the interactions with a good effect (Figure S2). We confirmed that the haploid chromosome numbers were ten based on microscopic observations of basidia of the re-synthesized dikaryon from the two monokaryons SP3 and SP30, with a confocal microscope (Figure S3). Furthermore, the Benchmarking Universal Single-Copy Orthologs (BUSCOs, basidiomycota_odb9) was used to assess assembly qualities. A total of 1335 BUSCOs were determined in the SP3 and SP30 genome assemblies, and the complete BUSCOs rates were 96.4% and 96.2%, respectively, for SP3 and SP30 (Table 2).

Repetitive sequences represent approximately 31.35% and 31.28% of the SP3 and SP30 genomes, respectively (Table S2). Most of the repeats belonged to long-terminal repeats (LTR), which were 25.58% and 24.99% of the SP3 and SP30 genomes, respectively (Table S2). The G + C content of the SP3 genome assembly was 46.00%, while in the SP30 assembly, it was 45.85%. In total, 11,455 and 11,245 protein-coding gene models were predicted for the SP3 and SP30 genomes, respectively. Approximately 44.03% and 44.33% of the gene models can be assigned to GO catalogs, 98.28% and 98.13% can be assigned to NR protein sequences, 60.70% and 61.37% can be assigned to InterProScan, and 31.34% and 31.33% can be assigned to KEGG pathways, respectively, for the SP3 and SP30 genomes (Table S3). The annotated non-coding RNAs were 1.25% and 1.56% of the SP3 and SP30 genomes, respectively (Table S4).

### 3.2. Collinearity Analysis of L. edodes Strains

**Figure 2:**
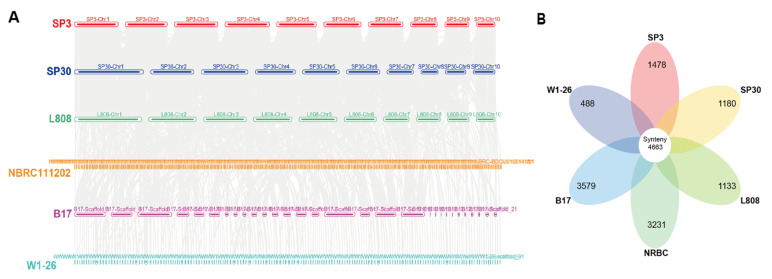
Collinearity analysis between the two monokaryotic genomes in a dikaryotic cultivar of *Lentinula edodes*. (**A**) Collinearity analysis between SP3 and SP30 genomes and the published genomes of *L. edodes* strains L808, NBRC111202, B17, and W1-26. (**B**) Flower diagram of core and specific genes in *L. edodes genomes*. The number in the center means synteny gene numbers, and numbers on the petal mean the specific gene number of each genome.

We downloaded the publicly available genome sequences and annotation information of four monokaryotic strains of *L. edodes* on NCBI (Table S5). The synteny genes among *L. edodes* strains were explored from collinearity analysis by MCSCAN, and the collinearity map among the *L. edodes* strains was drawn (Figure 2A). According to the results of collinearity analysis, strains SP3 and SP30 had a strong synteny relationship with strain L808, consistent with their close relationship to strain L808 [53]. The number of synteny genes among the six *L. edodes* strains was 4663 (Figure 2B). The genes of each strain that were not syntenic with any of the other five strains were recognized as strain-specific rearranged genes among *L. edodes* strains. Based on this criterion, the number of rearranged genes varied significantly among the six strains, with the smallest number of 488 for strain W1-26 and the largest number of over 3000 for strains NBRC111202 and B17. The number of rearranged genes in strains SP3, SP30, and L808 were approximately 1000 for each. 

**Figure 3:**
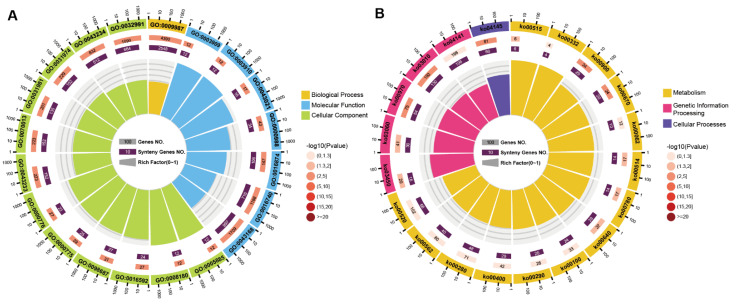
KEGG and GO cluster of syntenic genes between the two monokaryotic genomes in a dikaryotic cultivar of *Lentinula edodes*. (**A**) GO term analysis of synteny genes. Yellow label belongs to biological process class; blue label belongs to molecular function class; green label belongs to cellular component class. (**B**) KEGG term analysis of synteny genes. Yellow label belongs to metabolism class; magenta label belongs to genetic information processing class; purple label belongs to cellular process class. The outer ring indicates the top 20 GO or KEGG terms and the number of genes indicated on the outer circle. The second ring indicates the number of the genes in the genome background and *p*-values for the enrichment of synteny genes for the specified biological process. The inner ring indicates the number of synteny genes. The fourth circle indicates the enrichment factor of each GO or KEGG term.

The syntenic genes of *L. edodes* were analyzed by GO and KEGG for functional enrichment. According to GO analysis, the synteny genes were significantly enriched in cellular process, ribonucleoprotein complex binding, chromosomal structural proteins, kinetochore, nuclear lumen, etc., mostly related to cellular components (Figure 3A; Table S6). The results of KEGG analysis showed that the syntenic genes were significantly enriched in terpenoid backbone biosynthesis, one carbon pool by folate, propanoate metabolism, ribosome, phagosome, etc., that are all related to fundamental metabolism (Figure 3B; Table S7).

### 3.3. Comparative Analysis of SP3 and SP30

According to the genome multiple synteny plot, the chromosomes of strains SP3 and SP30 showed large segments of rearrangements between each other (Figure S4). For example, chromosomes 1 and 2 in strain SP3 were correlated with parts of chromosomes 1 and 9 of strain SP30. Although chromosome numbers of SP3 and SP30 strains were both 10, there were great differences in the length of their homologous chromosomes. The longest chromosome in strain SP3 was chromosome 4, with a length of 6.14 Mb, while the longest chromosome in strain SP30 was chromosome 1, with a length of 8.94 Mb (Table S1). Collinearity analysis showed that 8526 gene pairs were syntenic between SP3 and SP30. In the SP3 genome, 3136 protein-coding genes were not collinear with SP30, and 2921 such genes were not collinear with the SP3 genome in strain SP30. These rearranged genes were broadly distributed across chromosomes within each of the two strains (Figure S6). 

**Figure 4:**
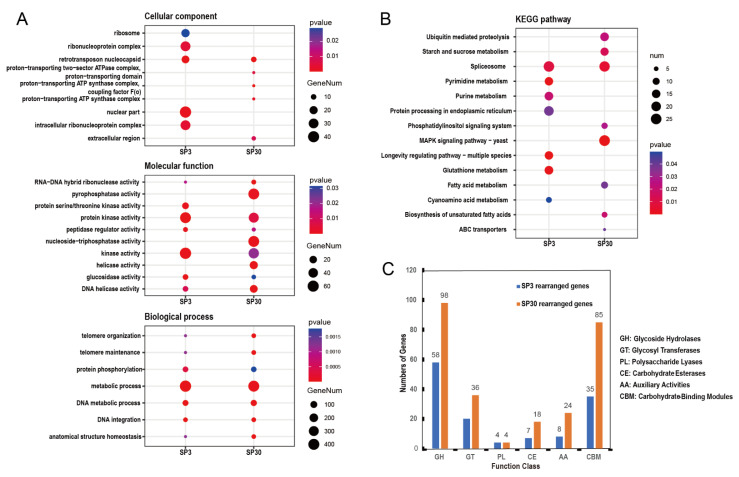
Cluster analysis of the two monokaryon-specific genes in each of the two monokaryons SP3 and SP30. (**A**) GO term analysis of SP3 and SP30 rearranged genes (top five terms of both SP3 and SP30). (**B**) KEGG term analysis of SP3 and SP30 rearranged genes (*p* < 0.05). (**C**) Classification of identified CAZymes. In GO and KEGG analysis, the color represents the *p*-value, the red color depth *p*-value was less than 0.01, and the blue color depth *p*-value was less than 0.05. The point size signifies the numbers of SP3 and SP30 rearranged genes.

We performed functional enrichment analysis of these rearranged genes within each of the two genomes. According to the classification of the identified CAZymes, the number of CAZymes of SP30 rearranged genes was much higher than that in strain SP3 (Figure 4C; Table S8). In particular, glycoside hydrolases (GH) and carbon binding modules (CMB) of SP30 rearranged genes were much enriched, with the numbers being almost twice those in strain SP3. Based on the GO cluster results, the SP3 rearranged genes were particularly enriched in retrotransposon nucleocapsid, nuclear components, and the intracellular ribonucleoprotein complex (Figure 4A; Table S9). By comparison, GO results showed that SP30 rearranged genes were particularly enriched in pyrophosphatase activity, nucleoside–triphosphatase activity, and helicase activity (Figure 4A; Table S9). KEGG results showed that the SP3 rearranged genes were particularly enriched in pyrimidine metabolism, purine metabolism, protein processing in endoplasmic reticulum pathways, longevity regulation, and glutathione metabolism (Figure 4B; Table S10). In contrast, for strain SP30, KEGG results showed that its rearranged genes were particularly enriched in ubiquitin-mediated proteolysis, starch and sucrose metabolism, MAPK signaling pathway, biosynthesis of unsaturated fatty acids, phosphatidylinositol signaling system, fatty acid metabolism, and ABC transporters (Figure 4B; Table S10).

**Figure 5:**
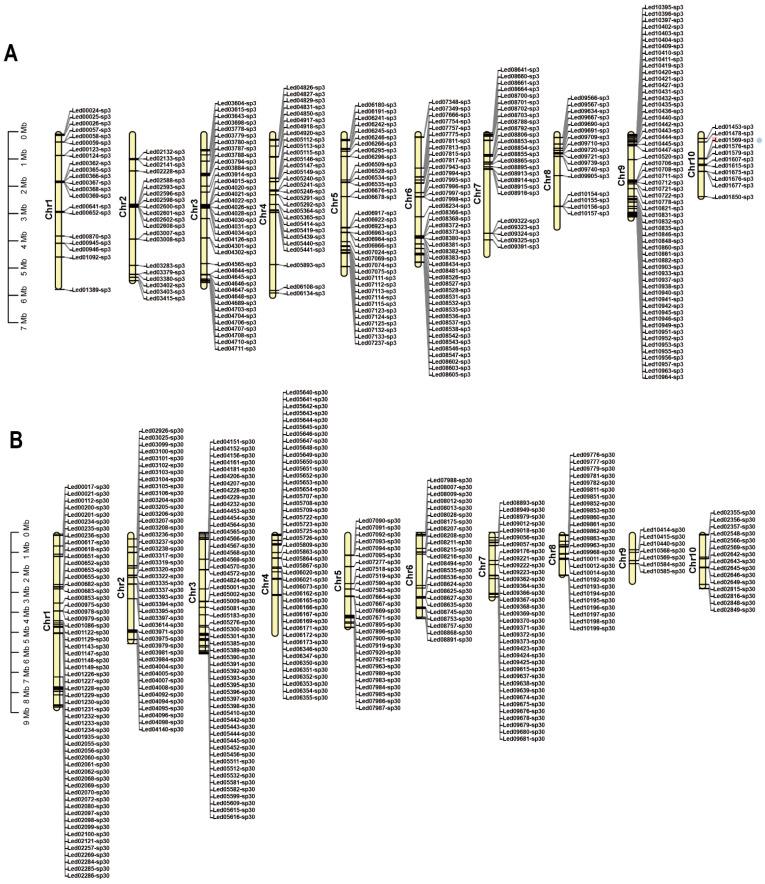
Location of the unique genes found within each of the two monokaryons on each chromosome. (**A**) Unique genes in strain SP3. (**B**) Unique genes in strain SP30.

Among these rearranged genes in SP3 and SP30, about 10% were completely absent in the genome of the other strain. We defined these genes as unique genes of strains SP3 or SP30. Specifically, for strains SP3 and SP30, there were 333 and 366 unique genes in their genomes, respectively, that were absent in the other monokaryon. Interestingly, most of the unique genes in the SP3 genome were distributed on chromosome 9. In the SP30 genome, the unique genes were mainly found on the first, second, and third chromosomes. Moreover, the unique genes were frequently found at the ends of chromosomes (Figure 5). Our PCR results confirmed that all the unique genes selected for confirmation were identified as unique genes in each of the two genomes (Figure S7; Table S11). However, among the genes selected for confirmation of their uniqueness, the PCR product size in SP30 amplified with primer pairs 02286.1-F and 02286.1-R was similar to that in SP3. To confirm their identities in these two strains, the PCR products of the amplified genes in both strains were sequenced. The Sanger sequencing results indicated that their DNA sequence identity was 15% for gene 02286 between SP3 and SP30 (Table S12). A similar result was obtained for gene 00368, but the DNA sequence identity between strains SP3 and SP30 was 63%. Except for these two genes, the presence/absence of gene products, as well as the size differences (in the case of successful amplification for both monokaryotic strains), for other genes was all consistent with expectations of them being unique genes between the two strains.

### 3.4. Analysis of Mating-Type Genes

**Figure 6:**
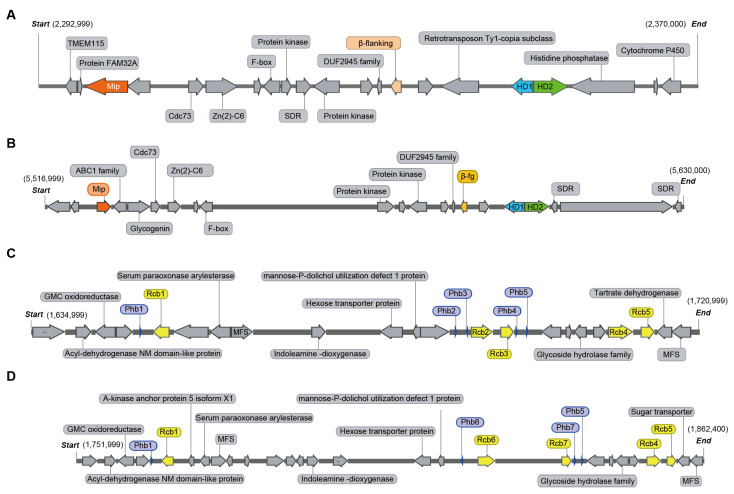
Distribution of genes in the matA and matB loci of the two monokaryons. (**A**) SP3 matA. (**B**) SP30 matA. (**C**) SP3 matB. (**D**) SP30 matB. Homeodomain (HD), pheromone receptor (RCB), pheromone (phb), mitochondrial intermediate peptidase gene (mip), β-flanking gene (β-fg).

As a species with a tetrapolar mating system, the *L. edodes* genome is expected to contain two unlinked mating-type loci, A and B [8]. The homeodomain (HD) transcription factors and the flanking genes at the matA locus are highly conserved in most mushroom-forming fungi. A previous study identified that the matA locus contained HD1 and HD2 in *L. edodes*, with the two genes separated by an approximately 210-bp-long intergenic region and transcribed in opposite directions [54]. In this study, we also found HD1, and HD2 homeodomain transcription factors located adjacent to each other on Chr2 of strain SP3 and Chr1 of strain SP30 (Figure 1). However, another HD protein was identified on the same chromosome at a distance of about 2.8 Mb from the main HD region in both strains (Figure 1). The HD proteins in these two monokaryons shared a high amino acid sequence identity (>81.65%) with four other strains, L808, B17, NBRC111202, and W1-26, of *L. edodes* (Figure S8). Consistent with previous observations, the mitochondrial intermediate peptidase gene (*mip*) and beta-flanking gene (*β-fg*) are both located on the same chromosome as the matA locus. However, there were differences in both distance and gene structure from *mip* (distance was about 45 kb in SP3 and 69 kb in SP30) and *β-fg* (distance was about 12.9 kb in SP3 and 6.8 kb in SP30) to HD1 between strains SP3 and SP30 (Figure 6A,B).

Unlike the matA locus that contains HD transcription factors, the matB locus contains pheromone receptor (*rcb*) and pheromone (*phb*) genes. In this study, B mating-type genes were located on Chr3 of both strains SP3 and SP30. The matB locus in strain SP3 contained five pheromone receptors and five pheromone genes, while in strain SP30, the matB locus also contained five pheromone receptors but only four pheromone genes (Figure 6C,D). Similar to previous studies [8], subloci Bα and Bβ (*rcb*2 and *rcb*3 of SP3, and rcb6 and rcb7 of SP30) were found together with their associated *phbs*, which determined their mating specificity. In contrast, the non-mating-type receptors (*rcb*4 and *rcb*5) were highly homologous between SP3 and SP30 and were devoid of associated *phb* genes (Figure 6C,D). Similar to the genome of the W1-26 strain, we also found a third sub locus in matB, where *rcb* was associated with *phb*, but where both the *rcb* and *phb* genes were highly homologous between SP3 and SP30 (Figure 6C,D). Furthermore, *rcbs* were also identified on another chromosome of both the SP3 and SP30 genomes but without an associated *phb* within a 20 kb flanking region, consistent with results in a previous study [8]. Overall, strains SP3 and SP30 showed differences in the number and locations of *rcbs* (Figure 1).

## 4. Discussion

In this study, we used Illumina and HiFi sequencing in combination with Hi-C assisted technology to assemble a pair of haploid genomes from the monokaryons of a commercial *L. edodes* cultivar. Both monokaryotic genomes here were assembled to higher completions (Contigs N50 = 3.40 Mb and 2.64 Mb, respectively) than those reported previously for *L. edodes* (Contigs N50 = 2.16 Mb of strain L808, Contigs N50 = 0.85 Mb of strain B17, Contigs N50 = 0.44 Mb of strain NBRC111202, Contigs N50 = 0.10 Mb of strain W1-26). The whole genome sizes of strains SP3 and SP30 were estimated at 50.83 Mb and 49.80 Mb, respectively, both of which are higher than the previously released *L. edodes* genomes (L808 = 45.87 Mb, B17 = 46.11 Mb, W1-26 = 41.82 Mb). The large differences in estimated genome sizes were mainly due to the underestimates of repetitive elements in previously published genomes. For example, the proportion of repetitive sequences in strains SP3 and SP30 (31.35% and 31.28%) is much higher than those of the reported mushroom strains (e.g., 16.24% in strain W1-26). We followed the commonly used methods for detecting repeat sequences in fungal genomes by RepeatMasker and RepeatModeler. In addition, we performed a de novo search for LTR retrotransposons using LTR_FINDER and identified tandem repeats using the TRF package. De novo TE accounts for nearly 30% of each genome. Most of the differentially estimated repetitive elements belonged to LTR, where the proportions of LTRs in the genomes of SP3 and SP30 reached ~25% [55]. We grouped the LTRs into four superfamilies: Gypsy, Copia, Ngaro, and unknown. Nearly 80% of LTR belonged to the Gypsy family in both genomes (Table S13). However, other repetitive elements also contributed to the genome size differences. Transposons are an important source of genome size variation within and among many organisms [56]. We wish to note that if certain repetitive sequences were recently duplicated and tandemly located next to each other to form long tracks of repeated sequence distributed in different parts of the genome, they could cause chromosomal assembly mistakes which may result in erroneous calls of rearrangements and non-synteny. Targeted PCR around the putative rearrangement junctions is needed in order to confirm the putative rearrangements. Furthermore, analysis of meiotic progeny genotypes could shed light on the potential roles of rearranged regions on progeny viability and phenotypes.

In this study, we used collinearity to identify syntenic core genes among the six sequenced monokaryons of *L. edodes*. Studying the conserved genes among strains within *L. edodes* and mushroom-forming fungi can help us understand the fundamental genes and processes involved in the development of fungal multicellularity and mushroom fruiting body formation. The results here lay the foundation for future targeted analyses of developmental stage-specific transcriptome data to determine the relationships between genome organization, gene expression, and mushroom development in *L. edodes* and other mushrooms. The syntenic genes account for about 40% of protein-coding genes in the genomes of the six compared *L. edodes* strains. With GO enrichment analysis, the syntenic genes were mainly enriched in DNA ligase activity, Arp2/3 protein complex, COP9 signalosome, macromolecular complex, transferase activity, anion binding (Table S6). The results suggest that the cellular components and DNA ligase activity functions, etc., are relatively conserved among the *L. edodes* strains in their chromosomal locations. The KEGG enrichment results showed that the syntenic genes were significantly enriched in functional categories involved in cell wall synthesis (such as chitin synthase, 1,3-beta-glucan synthase, chitosanase, etc.), fungal cell wall remodeling, targeted protein degradation, signal transduction, adhesion, and small secreted proteins, all of which are part of the core genetic program regulating complex multicellularity and fruiting body development in Agaricomycetes [57].

One major difference between Agaricomycetes and other eukaryotes, such as animals and plants, is in their nuclear genetic material organizations. In most agaricomycetes, each cell of a fertile strain typically has two or more haploid nuclei through most of their life cycles, rather than a single diploid nucleus [58]. In this study, we compared the genome difference between two haploid nuclei of a dikaryotic cultivar. Our analyses identified significant differences between these two genomes, in their overall chromosome structural arrangements and in their gene contents (Figure S4). The chromosome structural arrangements between these two genomes were similar to that found between the haploid nuclei in the most widely grown commercial strain Horst U1 in *Agaricus bisporus* [59]. Similar chromosome structural arrangements have also been reported in another mushroom, *Pleurotus pulmonale* [60], and between the two divergent lineages in the human basidiomycete yeast *Cryptococcus neoformans* species complex [61]. In contrast, the collinearity between homologous chromosomes is much higher in plants and animals, such as *Oryza sativa* and *Zea mays* [62]. On the other hand, the number of unique genes in SP3 and SP30 were higher than those found between the haploid genomes in Horst U1 in *A. bisporus* and between the two serotypes in the *C. neoformans* species complex.

The SNPs and indels between the SP3 and SP30 genomes were broadly distributed across the chromosomes (Figure 1 and Figure S5). The average SNP density in homologous genes between SP3 and SP30 was approximately one SNP per 280 bases. However, similar to that in Horst U1 in *A. bisporus*, the SNP distributions between SP3 and SP30 were not completely uniform across the chromosomes [59]. Several areas had a very low SNP density, such as those located at one end of chromosomes 2 and 6 of strain SP3 and one end of chromosomes 9 and 10 of strain SP30 (Figure 1). Such low-density SNP regions were expected given that the two ancestors of the cultivar strain JZB2102217 were both cultivars in China. Our results suggest that these two ancestral cultivars of JZB2102217 shared some common genetic backgrounds. Similar analyses of haploid genomes of other cultivars could help resolve the relationships among cultivars of *L. edodes* and identify common alleles associated with superior quality/quantity of shiitake cultivars for future breeding purposes.

Similar to SNP distributions, the rearranged genes were also broadly distributed across chromosomes between the SP3 and SP30 genomes (Figure S6). Interestingly, even though there was no significant difference in the total number of genes belonging to the CAZyme family between the two genomes (Figure S9), the SP30 genome had a much higher number of rearranged genes in the CAZyme family than that in the SP3 genome. This was confirmed based on KEGG classification where strain SP30 was significantly more enriched in genes for starch and sucrose metabolism pathways, including 14 genes related to glucoamylase, alpha-amylase, endoglucanase, eta-glucosidase, alpha-trehalase, beta-glucosidase, etc. This result suggested that strain SP30 seemed more adept at hydrolyzing and using starch and sucrose substrates, such as those in the PDA medium. In addition, previous research has shown that localized cell wall synthesis and hyphal apical extension are strongly linked [63,64]. Glucan and chitin are the essential components of fungal cell walls with glucanases and chitinases playing important roles in cell wall remodeling. The CAZyme family in fungi includes glucanases and chitinases, such as GH5, GH16, GH55, GH71, GH128, and other families [65], with SP30 rearranged genes being significantly enriched in the above-mentioned GH families. In contrast, the genome of strain SP3 did not show such a pattern (Table S8). These results indicated that there are great differences between the two monokaryons in carbohydrate metabolism. The inferred genomic differences between these two strains were consistent with their growth patterns on PDA medium, with strain SP30 growing faster than strain SP3, while strain SP3 had denser aerial hyphae than strain SP30 (Figure S10). However, their growth rates and patterns may differ from those observed on PDA if a different medium and/or different incubation condition was used.

Aside from growth differences, rearranged genes were also differentially enriched between the two strains for other physiological processes. Through KEGG analysis, except the formation of spliceosomes, SP3 and SP30 exhibited significant difference in several pathways. For example, strain SP3-rearranged genes were significantly enriched in the purine metabolism pathway, which is associated with producing flavor substances 5′-nucleotides in *L. edodes* [6]. For example, *fad2* encoding omega-6 fatty acid desaturase is related to aroma production in mushrooms, and it was enriched in the SP30 genome [6]. Overall, the results suggested that monokaryons SP3 and SP30 were likely highly complementary to each other in substrate utilization and in secondary metabolite productions. The GO enrichment analysis showed a similar pattern between the two nuclei. However, the potential interactions between the two nuclei in their divisions of labor and the extent of cooperation on the growth and reproduction of *L. edodes* need further research.

In Basidiomycota, the mating-type genes are often the most direct representations of how two nuclei coordinate to complete the reproductive life cycle. For species that require mating between monokaryons with complementary alleles at the mating-type loci to complete their sexual reproduction, their complementarity is usually based on both the reciprocal exchange of diffusible mating pheromones (P/R locus) and the interactions between the homeodomain proteins (HD) after cell fusion [66]. However, there are often several pairs of HD and P/R loci within individual genomes in Basidiomycota. In *Coprinopsis cinerea,* studies have reported that the HD locus is multiallelic and composed of two subloci [67,68]. However, different alleles appearing at only one sublocus is usually sufficient to induce the development and maintenance of the dikaryon. Therefore, the exact number of HD and P/R pairs can vary among strains within the same species [66]. For example, in *Flammulina velutipes*, the HD loci include subloci HD-a and HD-b, with a distance of more than 70 Kb [69]. However, the HD-a locus of *F. velutipes* is incomplete, containing only one HD2 domain and without any HD1 domain [70,71]. In this study, we found a new HD gene which contained the same domain as HD2 in *L. edodes*, at more than 2 Mb away from the complete HD locus. However, within both SP3 and SP30, the length of this new HD gene was only one quarter of that of the HD2 gene. Therefore, this extra HD2 gene was likely degenerated. Interestingly, in addition to the extra HD locus, there was an incomplete P/R sublocus with high homology to the *rcb* and *phb* genes in both SP3 and SP30. In this study, the six compared monokaryons were all from cultivated *L. edodes* strains. Whether all *L. edodes* strains have similar patterns is unknown at present. Understanding the emergence of the incomplete mating-type locus and its function could potentially help future breeding and cultivar maintenance efforts, such as reducing strain degeneration.

*L. edodes* is a very popular cultivated mushroom in East Asia. East Asia is the center of diversity and origin of domestication for this mushroom, with an estimated 500 commercial cultivars in China alone. Our study here provided fine scale analyses of two haploid genomes within a cultivar and revealed certain regions of high similarity and other regions of significant divergence between these two and other sequenced genomes. Our approach and the information obtained here could help identify the chromosomal regions of conservation among cultivars from different geographic regions and ecological niches. Such information could guide future breeding programs for developing geographic-specific (e.g., northern vs. southern China) and ecological niche-specific (e.g., wood log vs. cotton seeds as substrates) commercial cultivars [72].

## 5. Conclusions

In this study, we sequenced and assembled the complete genomes of two monokaryons from a widely grown cultivar strain of *L. edodes* using Illumina, HiFi, and Hi-C technologies. While both genomes were assembled into 10 chromosomes, there were significant differences in chromosomal structure and gene contents between the two monokaryons SP3 and SP30. According to comparative genomic analysis, the proportion of strain rearranged genes was about 30% between these two genomes. In addition, the two genomes were enriched for different sets of genes and gene families, including those involved in carbohydrate metabolism, secondary metabolism, and spliceosome formation. Specifically, SP30 strain rearranged genes were significantly enriched for starch and sucrose metabolism genes, and SP30 grew faster on PDA medium than strain SP3. In contrast, SP3 strain rearranged genes were enriched for producing flavor substances 5′-nucleotides. Discovery of incomplete mating-type genes in locations outside of the main mating-type loci suggests potentially continued evolution of the mating system in *L. edodes*. Together, the high-quality genome assemblies and the hypotheses generated here should facilitate future research on several fronts, including (i) analyzing the relationships among cultivars, and between cultivars and wild strains; and (ii) understanding the interactions at the transcriptome level and proteome level between the partner nuclei within the same cell; and (iii) laying a foundation for the subsequent better analysis of the genome recombination of progeny.

## Figures and Tables

**Table 1:** Genome assemblies of two monokaryons of *Lentinula edodes*.

Contents	SP3	SP30
Numbers of Scaffold	116	82
Total Length (bp)	50,830,139	49,800,313
Scaffold N50 (bp)	5,320,140	5,235,283
Contig Num	124	90
Contigs N50 (bp)	3,399,954	2,637,355
Pseudo chromosome	10	10
Pseudo-chromosome total length (bp)	47,651,827	46,345,524
Pseudo-chromosome N50 length (bp)	5,320,140	5,235,283
Chromosome anchoring rate for contigs (%)	93.74	93.05
Chromosome anchoring rate for contigs longer than 100 Kb (%)	99.63	98.91

**Table 2:** BUSCO analysis results of the two *Lentinula edodes* genomes.

Term	SP3		SP30	
	BUSCO Number	Proportion (%)	BUSCO Number	Proportion (%)
Complete BUSCOs	1287	96.4	1285	96.2
Complete and single-copy BUSCOs	1257	94.2	1262	94.5
Complete and duplicated BUSCOs	30	2.2	23	1.7
Fragmented BUSCOs	27	2	27	2
Missing BUSCOs	21	1.6	23	1.8
Total BUSCO groups searched	1335	100	1335	100

## Data Availability

The *L. edodes* SP3 and SP30 genomes and annotations sets are available at the National Genomics Data Center, China National Center for Bioinformation, under BioProject ID PRJCA007678 (https://ngdc.cncb.ac.cn/bioproject/browse/PRJCA007678, accessed on 22 December 2021).

## Supplementary Materials

The following supporting information can be downloaded at https://www.mdpi.com/2309-608X/8/2/167/s1. Figure S1: Schematic diagram of the genome assembly process. Figure S2: Whole-genome Hi-C interaction map at 50 k resolution; Figure S3: 3D image of chromosome structure during cell fusion within the basidia of the dikaryon formed mating strains SP3 and SP30; Figure S4: Dot plot of collinearity between SP3 and SP30; Figure S5: The distribution of SNP across the SP3 and SP30 chromosomes; Figure S6: Locations of the two monokaryon strains’ rearranged genes on each chromosome; Figure S7: PCR verification of selected unique genes; Figure S8: Differences in new homeodomain transcription factors (HD) of six strains of *Lentinula edodes*; Figure S9: CAZymes classification; Figure S10: Colony morphology and growth differences between the two monokaryons and their mating product dikaryon 3 × 30; Table S1: Contig numbers and length of pseudo chromosome within each of the two sequenced monokaryons; Table S2: Statistics of repeat sequence types in each genome; Table S3: Summary statistics of functional gene annotations based on information from different databases; Table S4: Annotation statistics of non-protein coding RNA with each of the two genomes; Table S5: Information of publicly available genomes of four monokaryotic strains used in collinearity analyses; Table S6: Top 20 GO term analysis of synteny genes; Table S7: Top 20 KEGG term analysis of synteny genes; Table S8: CAZyme cluster genes that are not syntenic between strains SP3 and SP30; Table S9: GO term analysis of rearranged genes between SP3 and SP30; Table S10: KEGG term analysis of rearranged genes between SP3 and SP30; Table S11: Unique genes and primers for PCR analysis. Table S12: PCR and Sanger sequencing identity of the unique genes within each of two genomes. Table S13: The numbers of retrotransposons belonging to various long-terminal repeat (LTR) superfamilies in the two monokaryotic strains.
