# Correction: Gao et al. Haplotype-Resolved Genome Analyses Reveal Genetically Distinct Nuclei within a Commercial Cultivar of *Lentinula edodes*. *J. Fungi* 2022, *8*, 167

**DOI:** 10.3390/jof8050496

**Published:** 2022-05-11

**Authors:** Qi Gao, Dong Yan, Shuang Song, Yangyang Fan, Shouxian Wang, Yu Liu, Yu Huang, Chengbo Rong, Yuan Guo, Shuang Zhao, Wentao Qin, Jianping Xu

**Affiliations:** 1 Beijing Engineering Research Center for Edible Mushroom, Institute of Plant Protection, Beijing Academy of Agriculture and Forestry Sciences, 9 Shuguang Garden Zhonglu, Haidian District, Beijing 100097, China; gaoqi@hotmail.co.jp (Q.G.); songshuang@ipepbaafs.cn (S.S.); fanyy@pku.edu.cn (Y.F.); wangshouxian@ipepbaafs.cn (S.W.); liuyu@ipepbaafs.cn (Y.L.); huangyu@ipepbaafs.cn (Y.H.); rongchengbo@ipepbaafs.cn (C.R.); guoyuan@ipepbaafs.cn (Y.G.); zhaoshuang@ipepbaafs.cn (S.Z.); qinwentao@ipepbaafs.cn (W.Q.); 2 State Key Laboratory of Protein and Plant Gene Research, Peking-Tsinghua Center for Life Sciences, School of Advanced Agricultural Sciences, Peking University, Beijing 100871, China; 3 College of Agriculture and Food Engineering, Baise University, 21 Zhongshan Second Street, Youjiang District, Baise 533000, China; 4 Institute of Agri-food Processing and Nutrition, Beijing Academy of Agricultural and Forestry Sciences, Beijing 100097, China; 5 Department of Biology, McMaster University, Hamilton, ON L8S 4K1, Canada

In the original article [1], the information regarding the SP30 unique gene was not correct. The authors wish to make the following corrections to this paper, which was published in the *Journal of Fungi*. Figure 5 should be replaced with the version below.

Additionally, text errors in this article will be corrected.

In the Materials and Methods section, “and the Juicebox was applied to adjust chromosome construction manually (Figure S2)” should be corrected to: “and the Juicebox was applied to adjust chromosome construction manually (Figure S1)”.

In the Results section, “Hi-C-assisted assembly of both genomes confirmed the interactions with a good effect (Figure S1)” should be corrected to: “Hi-C-assisted assembly of both genomes confirmed the interactions with a good effect (Figure S2)”.

In the Discussion section, “The SNPs and indels between the SP3 and SP30 genomes were broadly distributed across the chromosomes (Figures 1, 3, 4 and S5)” should be corrected to: “The SNPs and indels between the SP3 and SP30 genomes were broadly distributed across the chromosomes (Figures 1 and S5)”.

In Supplementary Materials, Table S9, “The highlight lines were presented in the Figure 3” should be corrected to: “The highlight lines were presented in the Figure 4”.

The addition of this correction does not change any of the statistics reported in the original analysis. All co-authors agree with the content of this correction and wish to apologize for any inconvenience to the readers resulting from these errors.

## Figures and Tables

**Figure 5:**
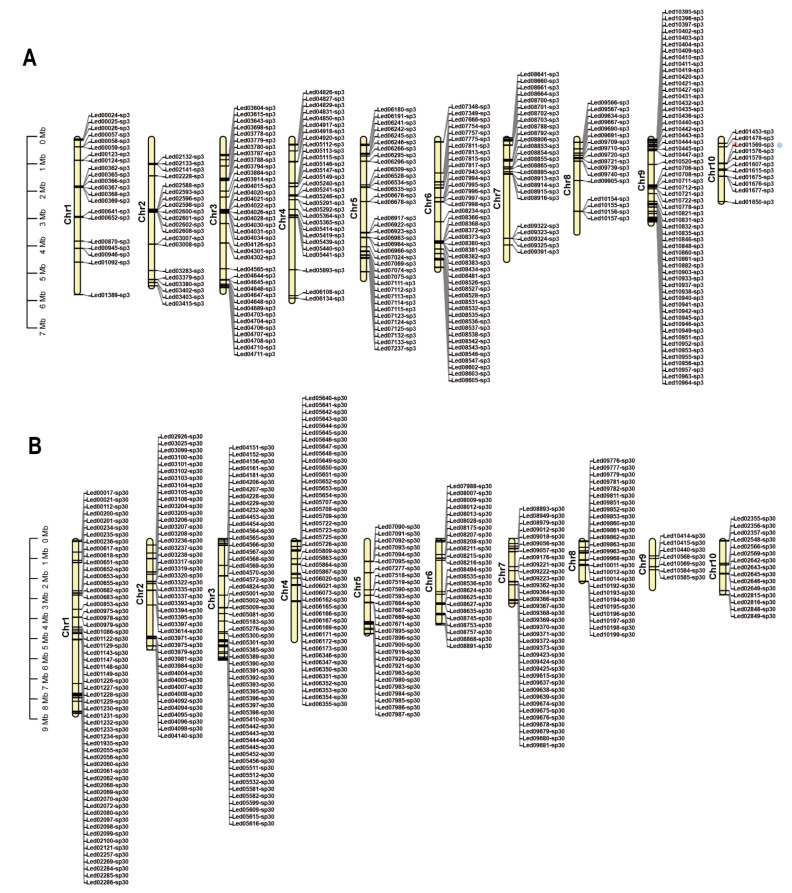
Location of the unique genes found within each of the two monokaryons on each chromosome. (**A**) Unique genes in strain SP3. (**B**) Unique genes in strain SP30.
