# Supplementary material for: Haplotype-Resolved Genome Analyses Reveal Genetically Distinct Nuclei within a Commercial Cultivar of Lentinula edodes
Source: J Fungi (Basel). 2022 Feb 9;8(2):167. doi: 10.3390/jof8020167 (PMC8877449; doi:10.3390/jof8020167)
Supplement: Supplementary file 1 [file jof-08-00167-s001.zip › jof-1556457-supplementary updated.pdf]

# Supplementary Materials

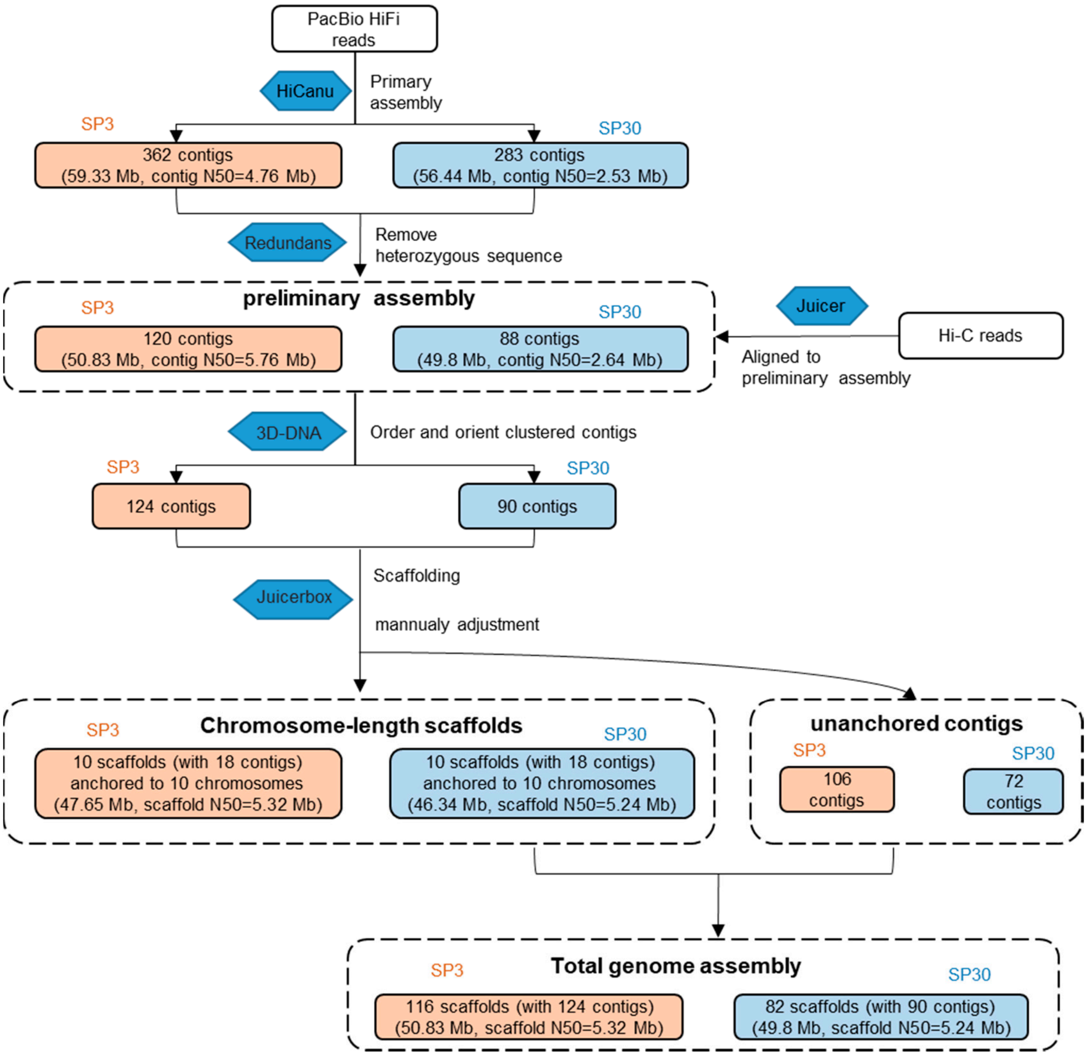


**Figure S1. Schematic diagram of the genome assembly process for strains SP3 and SP30.**

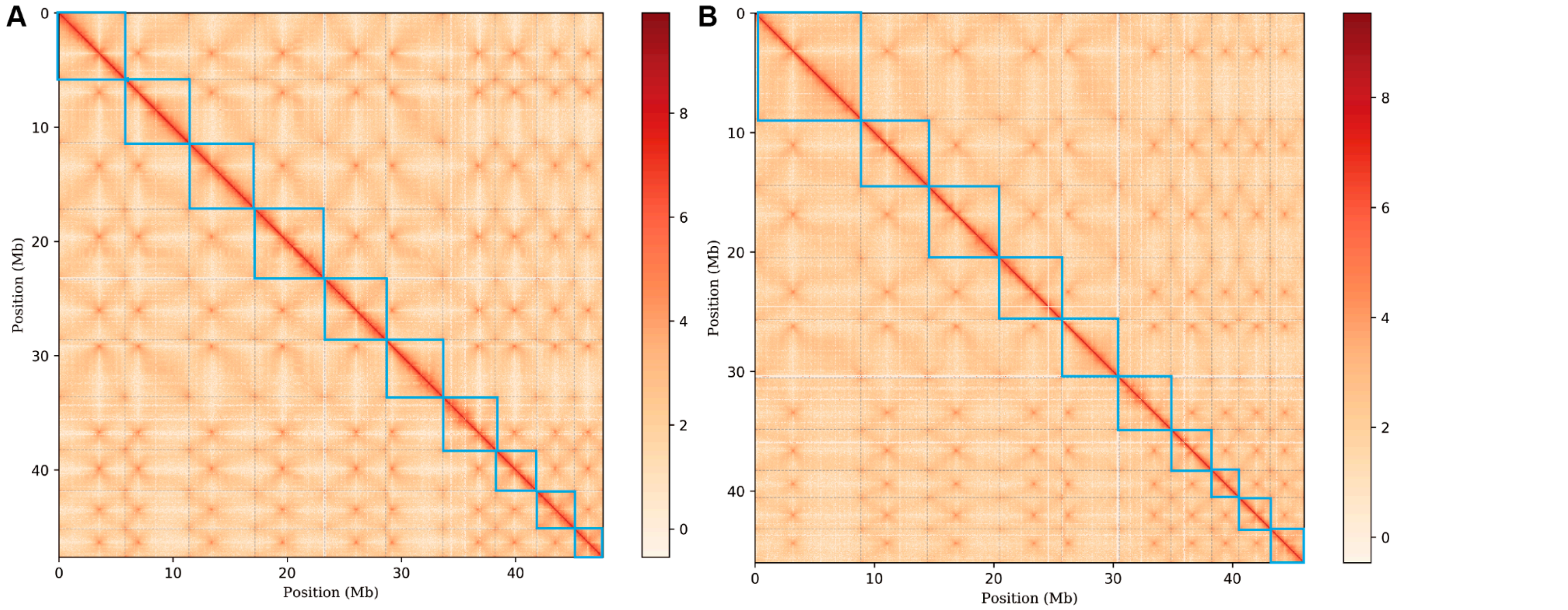


**Figure S2. Whole genome Hi-C interaction map at 50k resolution.** A: SP3. B: SP30. The color in the figure from lighter to darker indicates the increase in the intensity of the interaction, and the darker the stronger the interaction. The blue squares in the figure are the 10 chromosomes.

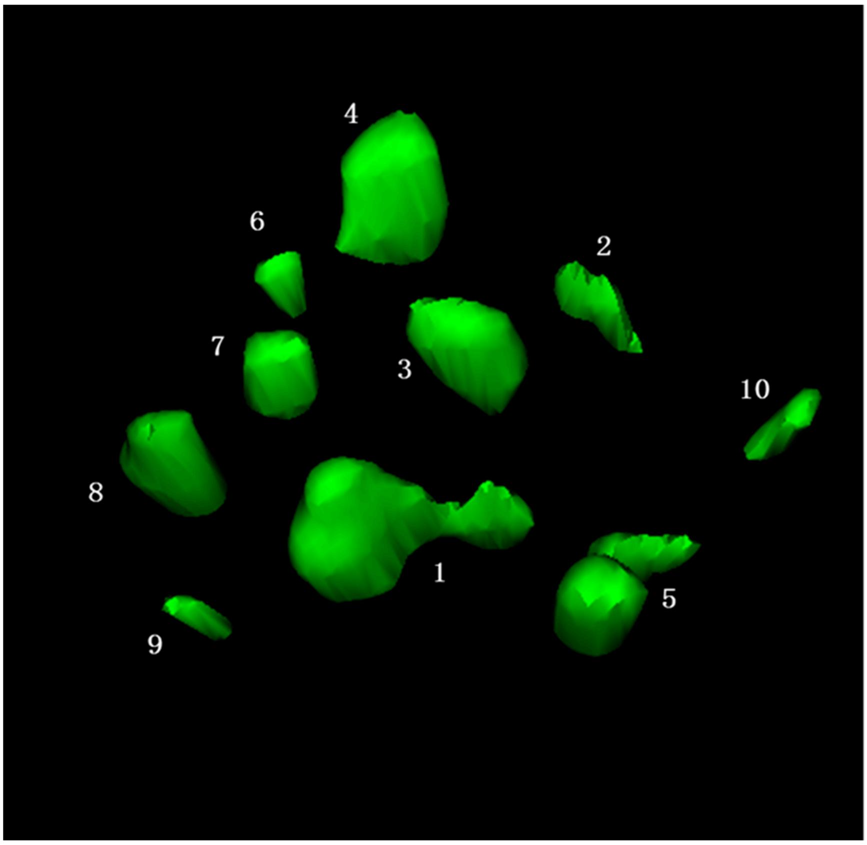


**Figure S3. 3D image of chromosome structure during cell fusion within the basidia of the dikaryon formed by mating strains SP3 and SP30.**

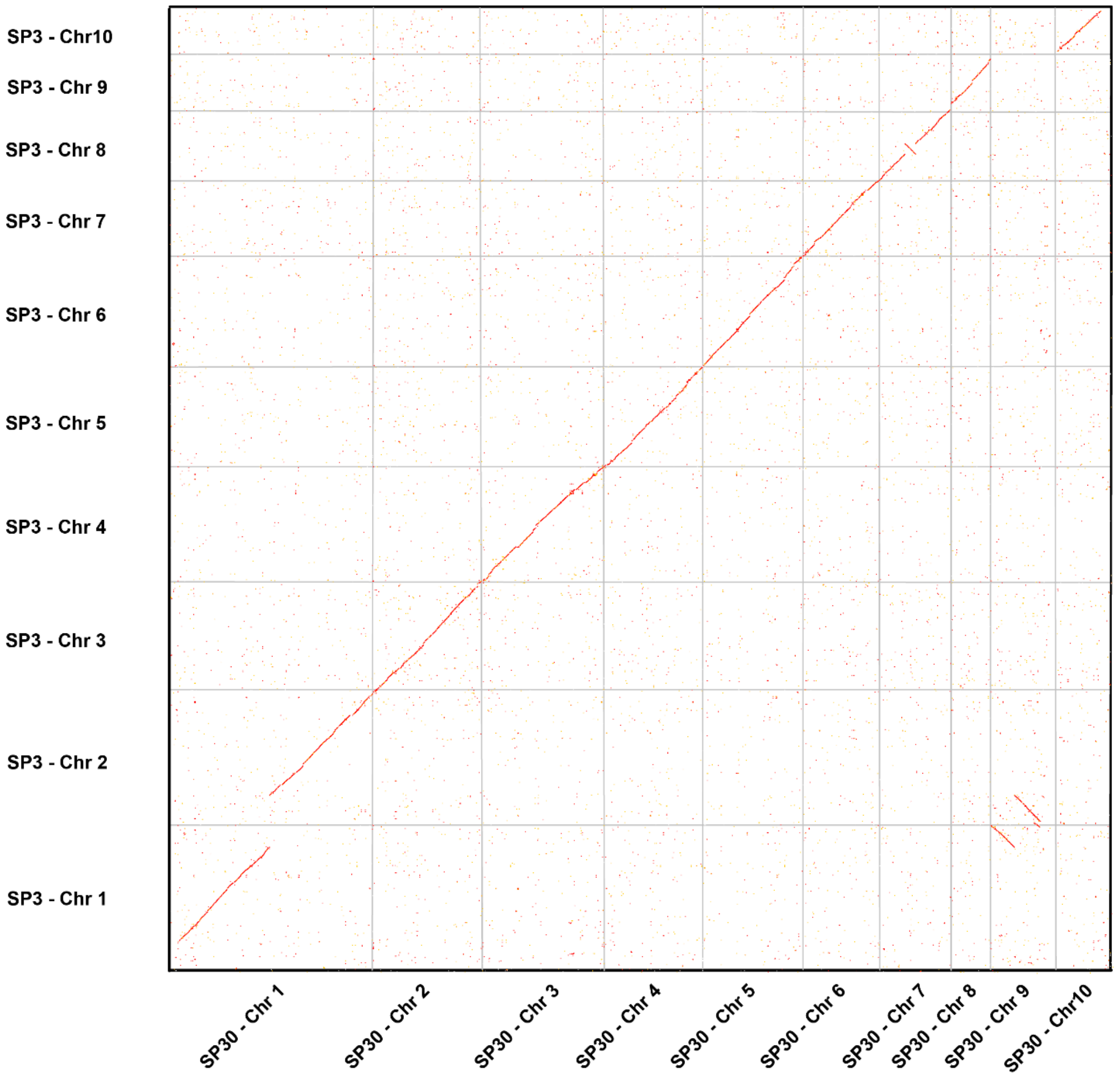


**Figure S4. Dot plot of collinearity between the genomes of strains SP3 and SP30.**

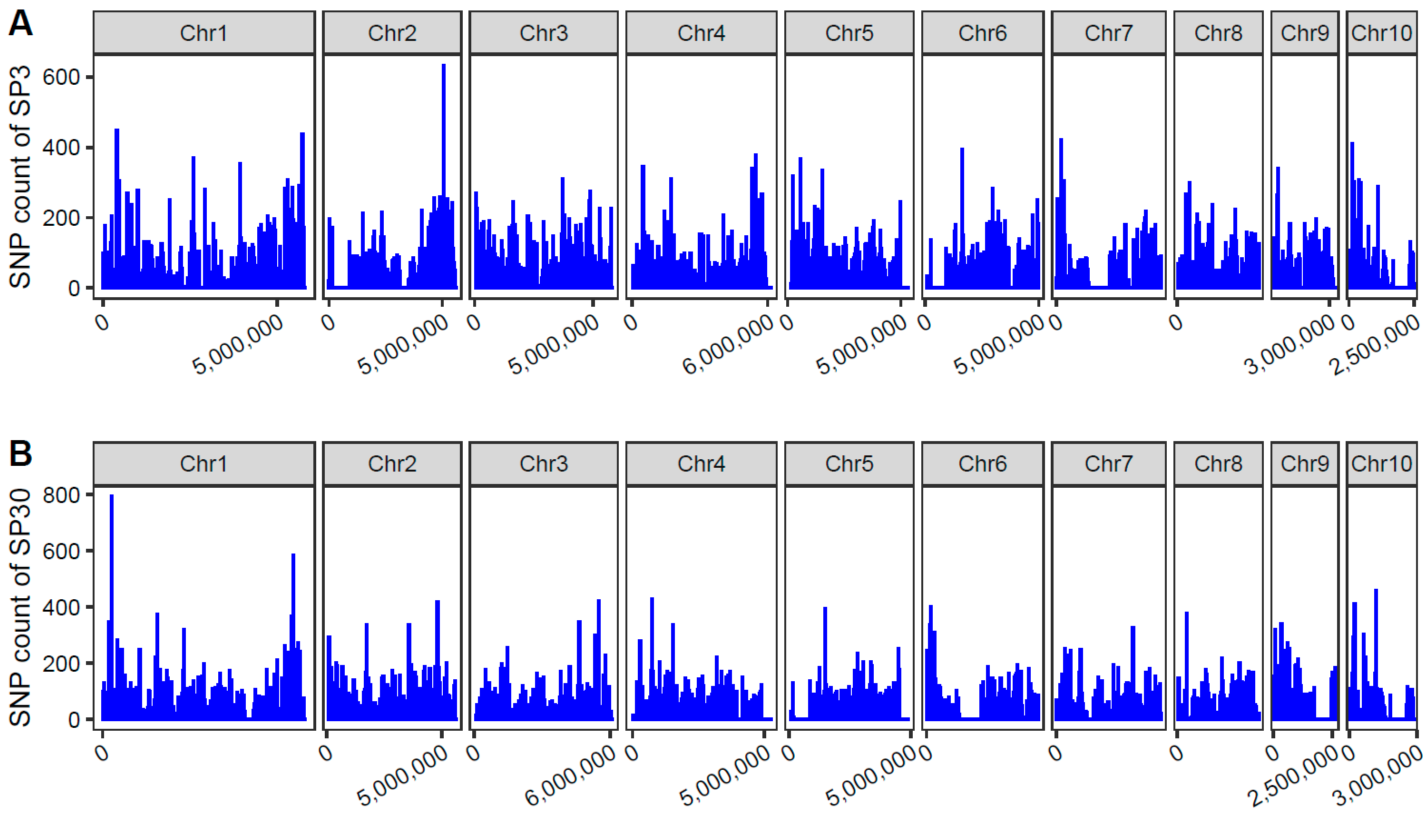


**Figure S5. The distribution of SNPs across the SP3 and SP30 chromosomes.** The X-axis represents chromosome length (bp), and the Y-axis represents the number of SNPs. Each bar represents the number of SNPs between homologous genes at 10kb sliding window.

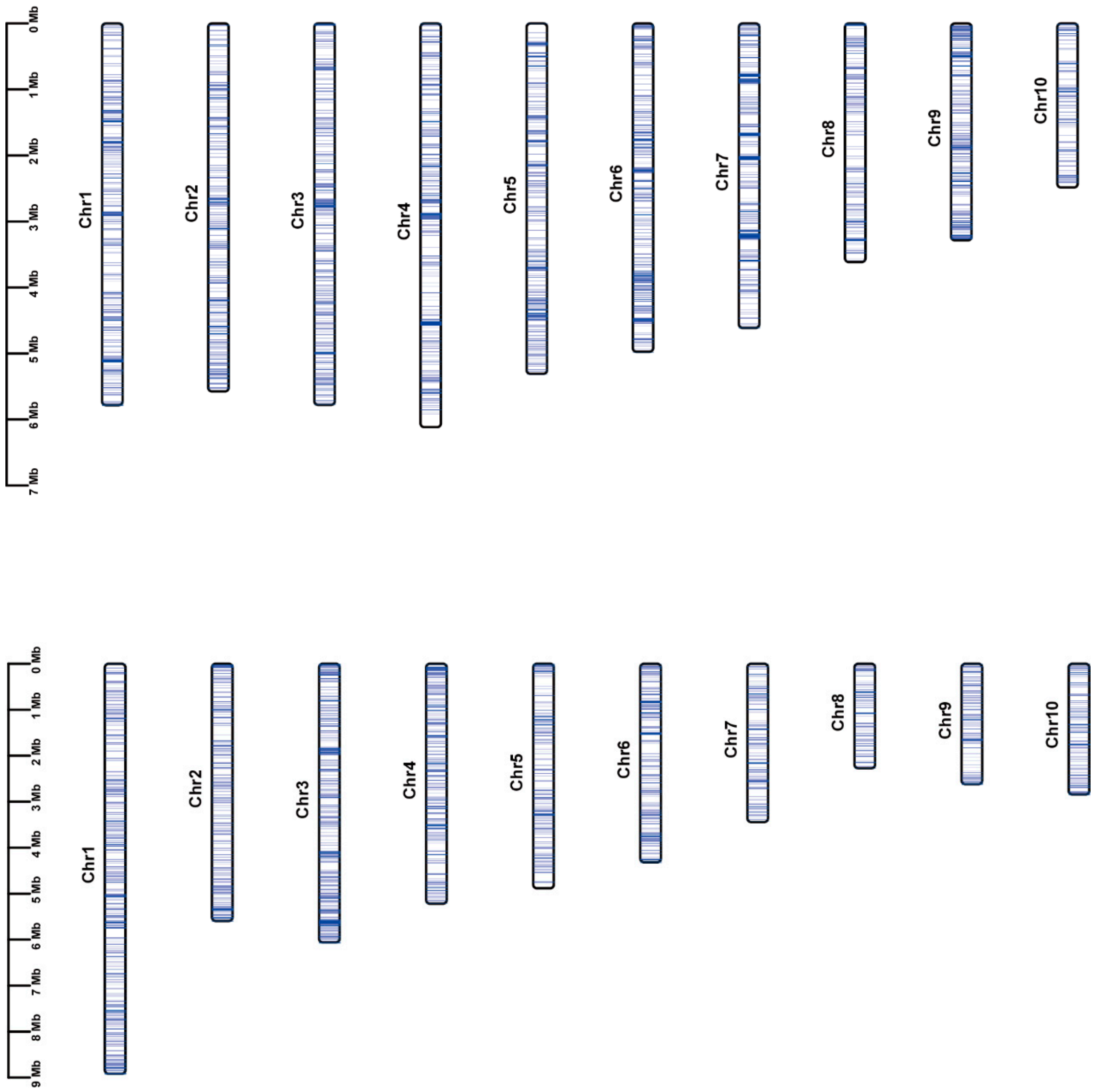

**Figure S6. Locations of the two monokaryons' rearranged genes on each chromosome.** A: Rearranged genes in the chromosomes of strain SP3. B: Rearranged genes in the chromosomes of strain SP30.

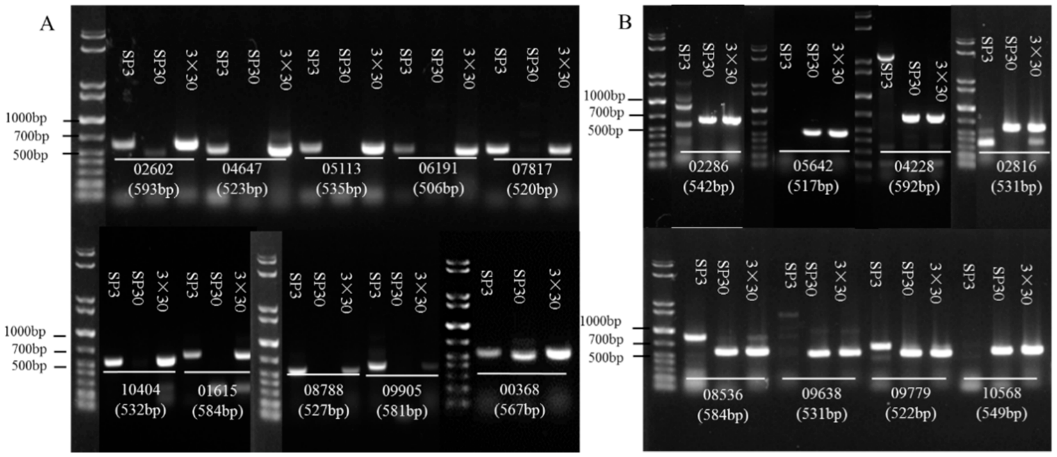


**Figure S7. PCR verification of selected unique genes.** A: Unique genes of SP3. B: Unique genes of SP30. Strain 3×30 is the dikaryon derived from mating between strains SP3 and SP30.

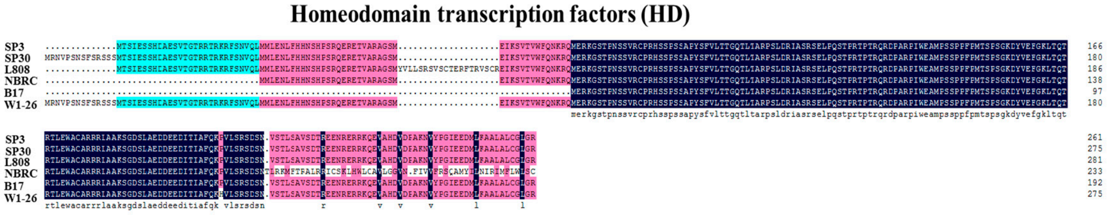


**Figure S8. Amino acid sequence alignment of the new homeodomain transcription factor (HD) among six monokaryotic strains of *Lentinula edodes*.**

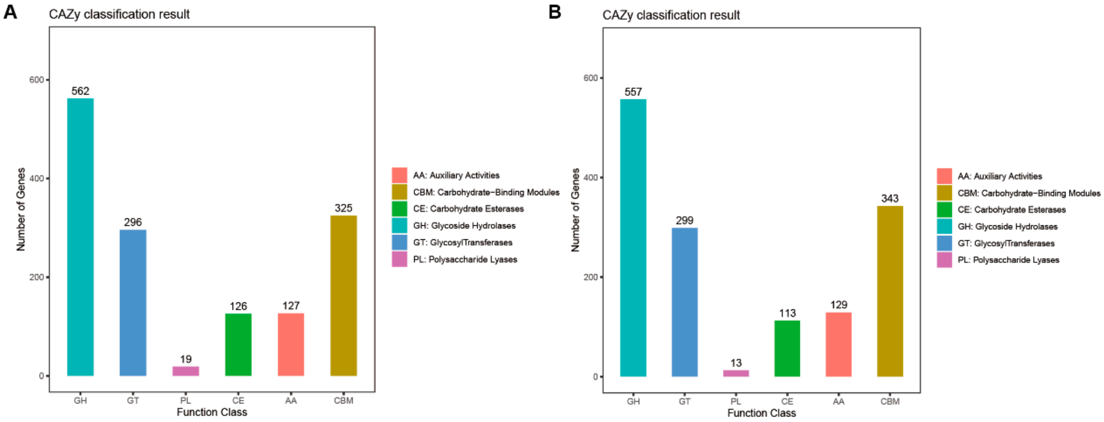


**Figure S9. Distributions of CAZymes within the genomes of strains SP3 and SP30. A: strain SP3. B: strain SP30.**

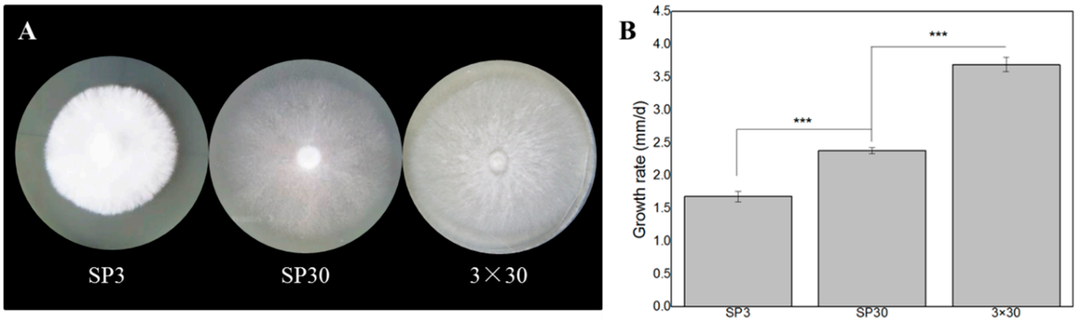


**Figure S10. Colony morphology and growth differences between the two monokaryons and their mating product dikaryon 3x30. A: Colony morphology developed after 12 days of growth on PDA; B: Radial growth rate differences among the three strains on PDA.**

**Table S1. Contig numbers and length of (pseudo-)chromosomes within each of the two sequenced strains.**

| Chromosome name | Number of Contigs | Length of Super-scaffold (bp) | Chromosome name | Number of Contigs | Length of Super-scaffold (bp) |
|---|---|---|---|---|---|
| **SP3-Chr1** | 1 | 5,795,191 | **SP30-Chr1** | 1 | 8,935,014 |
| **SP3-Chr2** | 2 | 5,586,483 | **SP30-Chr2** | 2 | 5,610,796 |
| **SP3-Chr3** | 2 | 5,791,229 | **SP30-Chr3** | 1 | 6,077,720 |
| **SP3-Chr4** | 3 | 6,127,555 | **SP30-Chr4** | 3 | 5,235,283 |
| **SP3-Chr5** | 2 | 5,320,140 | **SP30-Chr5** | 1 | 4,901,421 |
| **SP3-Chr6** | 2 | 4,985,548 | **SP30-Chr6** | 3 | 4,334,663 |
| **SP3-Chr7** | 2 | 4,624,895 | **SP30-Chr7** | 2 | 3,460,111 |
| **SP3-Chr8** | 1 | 3,626,384 | **SP30-Chr8** | 1 | 2,291,016 |
| **SP3-Chr9** | 2 | 3,297,434 | **SP30-Chr9** | 1 | 2,637,355 |
| **SP3-Chr10** | 1 | 2,496,968 | **SP30-Chr10** | 3 | 2,862,145 |
| **TOTAL** | 18 | 47,651,827 | **TOTAL** | 18 | 46,345,524 |

**Table S2. Statistics of repeat sequence types in each genome**

| | Type | RepeatMasker TEs Length (Bp) | RepeatMasker TEs % in genome | RepeatProteinMask TEs Length (Bp) | RepeatProteinMask TEs % in genome | De novo Length (Bp) | De novo % in genome | Repeat Finder (TRF) Length (Bp) | Repeat Finder (TRF) % in genome | Combined TEs Length (Bp) | Combined TEs % in genome |
|---|---|---|---|---|---|---|---|---|---|---|---|
| SP3 | DNA | 8,697 | 0.02 | 34,162 | 0.07 | 478,438 | 0.94 | 0 | 0 | 511,505 | 1.01 |
| | LINE | 7,136 | 0.01 | 79,425 | 0.16 | 331,080 | 0.65 | 0 | 0 | 380,201 | 0.75 |
| | SINE | 3,348 | 0.01 | 0 | 0 | 205,340 | 0.4 | 0 | 0 | 207,438 | 0.41 |
| | LTR | 1,077,132 | 2.12 | 4,389,225 | 8.63 | 12,833,588 | 25.25 | 0 | 0 | 13,004,473 | 25.58 |
| | Tandem repeats | 2,120 | 0 | 0 | 0 | 86,376 | 0.17 | 734,303 | 1.44 | 822,799 | 1.61 |
| | Unknown | 515 | 0 | 0 | 0 | 1,721,271 | 3.39 | 0 | 0 | 1,721,786 | 3.39 |
| | Total TE | 1,098,336 | 2.16 | 4,502,182 | 8.86 | 15,426,739 | 30.35 | 734,303 | 1.44 | 15,937,806 | 31.35 |
| SP30 | DNA | 8,664 | 0.02 | 49,476 | 0.1 | 528,975 | 1.06 | 0 | 0 | 576,412 | 1.16 |
| | LINE | 9,787 | 0.02 | 97,664 | 0.2 | 420,114 | 0.84 | 0 | 0 | 455,977 | 0.92 |
| | SINE | 3,283 | 0.01 | 0 | 0 | 39,446 | 0.08 | 0 | 0 | 41,907 | 0.08 |
| | LTR | 975,268 | 1.96 | 4,115,631 | 8.26 | 12,341,153 | 24.78 | 0 | 0 | 12,448,254 | 24.99 |
| | Tandem repeats | 2,214 | 0.01 | 0 | 0 | 137,405 | 0.27 | 754,178 | 1.51 | 893,797 | 1.79 |
| | Unknown | 352 | 0 | 0 | 0 | 1,817,603 | 3.65 | 0 | 0 | 1,817,955 | 3.65 |
| | Total TE | 999,333 | 2.01 | 4,262,771 | 8.56 | 15,157,417 | 30.43 | 754,178 | 1.51 | 15,580,460 | 31.28 |

**Table S3. Summary statistics of functional gene annotations based on information from different databases**

| | SP3 | | SP30 | |
|---|---|---|---|---|
| | Number | Percent(%) | Number | Percent(%) |
| **Total** | 11,455 | | 11,245 | |
| **InterPro** | 6,953 | 60.7 | 6,901 | 61 |
| **GO** | 5,044 | 44.03 | 4,985 | 44 |
| **KEGG_ALL** | 9,839 | 85.89 | 9,673 | 86 |
| **KEGG_KO** | 3,590 | 31.34 | 3,523 | 31 |
| **Swissprot** | 5,744 | 50.14 | 5,739 | 51 |
| **TrEMBL** | 11,242 | 98.14 | 11,026 | 98 |
| **NR** | 11,258 | 98.28 | 11,035 | 98 |
| **Annotated** | 11,261 | 98.31 | 11,037 | 98 |
| **Unannotated** | 194 | 1.69 | 208 | 1.85 |

**Table S4. Annotation statistics for non-protein coding RNA within each of the two genomes.**

| | Type | | Copy | Average length(bp) | Total length(bp) | % of genome |
|---|---|---|---|---|---|---|
| SP3 | tRNA | | 347 | 89 | 30,795 | 0.0606 |
| | rRNA | rRNA | 140 | 2,122 | 297,094 | 0.5844 |
| | | 18S | 44 | 1,836 | 80,776 | 0.1589 |
| | | 28S | 48 | 4,392 | 210,794 | 0.4147 |
| | | 5S | 48 | 115 | 5,524 | 0.0109 |
| | snRNA | snRNA | 63 | 77 | 4,830 | 0.0095 |
| | | CD-box | 47 | 57 | 2,683 | 0.0053 |
| | | splicing | 16 | 134 | 2,147 | 0.0042 |
| SP30 | tRNA | | 412 | 86 | 35,586 | 0.0715 |
| | rRNA | rRNA | 169 | 2,157 | 364,515 | 0.7319 |
| | | 18S | 58 | 1,874 | 108,679 | 0.2182 |
| | | 28S | 55 | 4,534 | 249,394 | 0.5007 |
| | | 5S | 56 | 115 | 6,442 | 0.0129 |
| | snRNA | snRNA | 73 | 73 | 5,364 | 0.0108 |
| | | CD-box | 58 | 57 | 3,313 | 0.0067 |
| | | splicing | 15 | 137 | 2,051 | 0.0041 |

**Table S5. Information of publicly available genomes of four monokaryotic strains used in collinearity analyses.**

| Strain | Isolate | BioSample | Submission | Sequence method | Level | Size (Mb) | GC% | Scaffolds | Contig N50 (Mb) | GenBank FTP |
|---|---|---|---|---|---|---|---|---|---|---|
| L808 | monokaryon | SAMN14591202 | China | PacBio Sequel | Scaffold | 45.8703 | 46.0791 | 25 | 2.16 | ftp://ftp.ncbi.nlm.nih.gov/genomes/all/GCA/015/476/405/GCA_015476405.1_ASM1547640v1 |
| B17 | monokaryon | SAMN04457462 | Korea | PacBio; Illumina HiSeq | Scaffold | 46.1067 | 45.9 | 31 | 0.85 | ftp://ftp.ncbi.nlm.nih.gov/genomes/all/GCA/001/562/095/GCA_001562095.1_B17 |
| W1-26 | monokaryon | SAMN03418238 | China | Illumina HiSeq | Scaffold | 41.8187 | 46.1 | 335 | 0.1 | ftp://ftp.ncbi.nlm.nih.gov/genomes/all/GCA/001/700/625/GCA_001700625.1_ASM170062v1 |
| NBRC 111202 | monokaryon | SAMD00054105 | Japan | Illumina Hiseq 2500 | Scaffold | 39.9229 | 44.7 | 1951 | 0.44 | ftp://ftp.ncbi.nlm.nih.gov/genomes/all/GCA/002/003/045/GCA_002003045.1_Lened_assembly01 |

**Table S6. Top 20 GO term analysis of synteny genes**

| Class | ID | Description | P value | Fg_num | Bg_num | Ratio |
|---|---|---|---|---|---|---|
| Biological Process | GO:0009987 | cellular process | 5.75E-05 | 2548 | 4390 | 0.58 |
| Molecular Function | GO:0043021 | ribonucleoprotein complex binding | 0.000549538 | 16 | 17 | 0.941 |
| Molecular Function | GO:0043168 | anion binding | 0.000663945 | 997 | 1709 | 0.583 |
| Molecular Function | GO:0003909 | DNA ligase activity | 0.000740123 | 12 | 12 | 1 |
| Molecular Function | GO:0003910 | DNA ligase (ATP) activity | 0.000740123 | 12 | 12 | 1 |
| Molecular Function | GO:0000988 | transcription factor activity, protein binding | 0.00083219 | 34 | 43 | 0.791 |
| Molecular Function | GO:0016874 | ligase activity | 1.12E-05 | 106 | 147 | 0.721 |
| Molecular Function | GO:0016740 | transferase activity | 0.000126033 | 716 | 1198 | 0.598 |
| Cellular Component | GO:0098687 | chromosomal region | 0.000358785 | 27 | 31 | 0.871 |
| Cellular Component | GO:0043233 | organelle lumen | 0.000367872 | 152 | 223 | 0.682 |
| Cellular Component | GO:0070013 | intracellular organelle lumen | 0.000367872 | 152 | 223 | 0.682 |
| Cellular Component | GO:0016592 | mediator complex | 0.000404134 | 24 | 27 | 0.889 |
| Cellular Component | GO:0000775 | chromosome, centromeric region | 0.000846118 | 25 | 29 | 0.862 |
| Cellular Component | GO:0005885 | Arp2/3 protein complex | 0.001237776 | 12 | 12 | 1 |
| Cellular Component | GO:0008180 | COP9 signalosome | 0.001237776 | 12 | 12 | 1 |
| Cellular Component | GO:0032991 | macromolecular complex | 0.0013853 | 664 | 1090 | 0.609 |
| Cellular Component | GO:0000776 | kinetochore | 0.00195659 | 23 | 27 | 0.852 |
| Cellular Component | GO:0031981 | nuclear lumen | 0.001991594 | 135 | 201 | 0.672 |
| Cellular Component | GO:0043234 | protein complex | 0.002050707 | 512 | 832 | 0.615 |
| Cellular Component | GO:0031974 | membrane-enclosed lumen | 0.002243533 | 152 | 229 | 0.664 |

## Table S7. Top 20 KEGG term analysis of synteny genes

| Class | ID | Description | P value | Fg_num | Bg_num | Ratio |
|---|---|---|---|---|---|---|
| Metabolism | ko00900 | Terpenoid backbone biosynthesis | 0.0005926 | 29 | 34 | 0.853 |
| Metabolism | ko00670 | One carbon pool by folate | 0.007766 | 20 | 24 | 0.833 |
| Metabolism | ko00640 | Propanoate metabolism | 0.0189288 | 28 | 37 | 0.757 |
| Metabolism | ko00514 | Other types of O-glycan biosynthesis | 0.0318249 | 14 | 17 | 0.824 |
| Metabolism | ko00780 | Biotin metabolism | 0.0318249 | 14 | 17 | 0.824 |
| Metabolism | ko00515 | Mannose type O-glycan biosyntheis | 0.0376213 | 6 | 6 | 1 |
| Metabolism | ko00100 | Steroid biosynthesis | 0.0577929 | 24 | 33 | 0.727 |
| Metabolism | ko00280 | Valine, leucine and isoleucine degradation | 0.0594712 | 48 | 71 | 0.676 |
| Metabolism | ko00062 | Fatty acid elongation | 0.0631628 | 10 | 12 | 0.833 |
| Metabolism | ko00400 | Phenylalanine, tyrosine and tryptophan biosynthesis | 0.0933658 | 29 | 42 | 0.69 |
| Metabolism | ko00520 | Amino sugar and nucleotide sugar metabolism | 0.0950497 | 66 | 102 | 0.647 |
| Metabolism | ko00290 | Valine, leucine and isoleucine biosynthesis | 0.1020119 | 20 | 28 | 0.714 |
| Metabolism | ko00562 | Inositol phosphate metabolism | 0.1043755 | 40 | 60 | 0.667 |
| Metabolism | ko00332 | Carbapenem biosynthesis | 0.1123555 | 4 | 4 | 1 |
| Genetic Information Processing | ko03010 | Ribosome | 0.0037207 | 130 | 193 | 0.674 |
| Genetic Information Processing | ko04141 | Protein processing in endoplasmic reticulum | 0.0656539 | 106 | 166 | 0.639 |
| Genetic Information Processing | ko03450 | Non-homologous end-joining | 0.0339706 | 16 | 20 | 0.8 |
| Genetic Information Processing | ko00970 | Aminoacyl-tRNA biosynthesis | 0.007792 | 54 | 75 | 0.72 |
| Genetic Information Processing | ko03060 | Protein export | 0.0314432 | 30 | 41 | 0.732 |
| Cellular Processes | ko04145 | Phagosome | 0.0016994 | 60 | 81 | 0.741 |

**Table S8. CAZyme cluster genes that are not syntenic between strains SP3 and SP30**

| CAZymes | SP3-special gene numbers | SP30-special gene numbers |
|---|---|---|
| GH | 58 | 98 |
| GT | 20 | 36 |
| PL | 4 | 4 |
| CE | 7 | 18 |
| AA | 8 | 24 |
| CBM | 35 | 85 |
| GH0 | 1 | 3 |
| GH1 | 1 | 1 |
| GH2 | 1 | 0 |
| GH3 | 5 | 7 |
| GH5 | 1 | 4 |
| GH6 | 1 | 2 |
| GH10 | 1 | 0 |
| GH11 | 1 | 1 |
| GH12 | 1 | 0 |
| GH13 | 2 | 3 |
| GH15 | 2 | 3 |
| GH16 | 2 | 7 |
| GH17 | 2 | 1 |
| GH18 | 4 | 11 |
| GH19 | 4 | 2 |
| GH23 | 0 | 1 |
| GH26 | 0 | 1 |
| GH27 | 0 | 3 |
| GH28 | 7 | 9 |
| GH29 | 0 | 1 |
| GH30 | 2 | 0 |
| GH31 | 2 | 2 |
| GH35 | 0 | 1 |
| GH36 | 0 | 1 |
| GH37 | 0 | 1 |
| GH38 | 3 | 2 |
| GH43 | 3 | 4 |
| GH45 | 0 | 1 |
| GH47 | 1 | 0 |
| GH55 | 1 | 3 |
| GH71 | 0 | 3 |
| GH75 | 2 | 1 |
| GH78 | 2 | 2 |
| GH79 | 0 | 3 |

| | | |
|---|---|---|
| GH92 | 0 | 2 |
| GH93 | 1 | 2 |
| GH95 | 1 | 1 |
| GH100 | 3 | 1 |
| GH105 | 0 | 1 |
| GH109 | 0 | 1 |
| GH128 | 0 | 3 |
| GH133 | 1 | 2 |
| GH134 | 0 | 1 |
| GT0 | 0 | 3 |
| GT1 | 8 | 7 |
| GT2 | 3 | 6 |
| GT4 | 2 | 4 |
| GT7 | 0 | 1 |
| GT8 | 0 | 1 |
| GT15 | 1 | 0 |
| GT20 | 0 | 1 |
| GT27 | 0 | 1 |
| GT31 | 1 | 0 |
| GT32 | 1 | 0 |
| GT34 | 1 | 2 |
| GT47 | 0 | 6 |
| GT50 | 0 | 2 |
| GT90 | 3 | 1 |
| GT92 | 0 | 1 |
| PL1 | 2 | 2 |
| PL4_2 | 2 | 2 |
| CE0 | 0 | 1 |
| CE4 | 1 | 4 |
| CE6 | 0 | 4 |
| CE8 | 2 | 2 |
| CE11 | 1 | 3 |
| CE12 | 1 | 0 |
| CE16 | 2 | 4 |
| AA1 | 0 | 2 |
| AA2 | 2 | 3 |
| AA3 | 3 | 11 |
| AA5 | 0 | 1 |
| AA7 | 1 | 2 |
| AA9 | 1 | 3 |
| AA10 | 1 | 2 |
| CBM1 | 7 | 12 |
| CBM2 | 3 | 6 |
| CBM5 | 0 | 1 |
| CBM6 | 2 | 5 |
| CBM13 | 4 | 16 |

| | | |
|---|---|---|
| CBM18 | 1 | 2 |
| CBM19 | 0 | 1 |
| CBM20 | 2 | 6 |
| CBM21 | 2 | 1 |
| CBM24 | 0 | 3 |
| CBM32 | 1 | 6 |
| CBM35 | 1 | 2 |
| CBM43 | 2 | 0 |
| CBM48 | 1 | 6 |
| CBM50 | 4 | 7 |
| CBM51 | 0 | 1 |
| CBM57 | 4 | 9 |
| CBM63 | 0 | 1 |
| CBM67 | 1 | 0 |

**Table S9. GO term analysis of rearranged genes between SP3 and SP30.**

The highlight lines were presented in the Figure 4.

| Genome | GO_class | GO ID | Description | Fg_num | Bg_num | Pvalue | Gene percent (%) |
|---|---|---|---|---|---|---|---|
| SP3 | Cellular component | GO:0000943 | retrotransposon nucleocapsid | 18 | 21 | 3.1E-13 | 85.71 |
| | Cellular component | GO:0044428 | nuclear part | 42 | 188 | 0.00101 | 22.34 |
| | Cellular component | GO:0030529 | intracellular ribonucleoprotein complex | 31 | 136 | 0.00363 | 22.79 |
| | Cellular component | GO:1990904 | ribonucleoprotein complex | 31 | 137 | 0.0041 | 22.63 |
| | Cellular component | GO:0005840 | ribosome | 21 | 97 | 0.02861 | 21.65 |
| | Cellular component | GO:0043228 | non-membrane-bounded organelle | 41 | 225 | 0.04739 | 18.22 |
| | Cellular component | GO:0043232 | intracellular non-membrane-bounded organelle | 41 | 225 | 0.04739 | 18.22 |
| | Molecular function | GO:0004672 | protein kinase activity | 57 | 214 | 0.00012 | 26.64 |
| | Molecular function | GO:0016301 | kinase activity | 63 | 251 | 0.00034 | 25.10 |

| | | | | | | |
|---|---|---|---|---|---|---|
| Molecular function | GO:0015926 | glucosidase activity | 9 | 16 | 0.00037 | 56.25 |
| Molecular function | GO:0004674 | protein serine/threonine kinase activity | 15 | 39 | 0.00095 | 38.46 |
| Molecular function | GO:0061134 | peptidase regulator activity | 6 | 9 | 0.00115 | 66.67 |
| Molecular function | GO:0016773 | phosphotransferase activity, alcohol group as acceptor | 58 | 241 | 0.00175 | 24.07 |
| Molecular function | GO:0020037 | heme binding | 39 | 148 | 0.00178 | 26.35 |
| Molecular function | GO:0046906 | tetrapyrrole binding | 39 | 148 | 0.00178 | 26.35 |
| Molecular function | GO:0004470 | malic enzyme activity | 5 | 7 | 0.00204 | 71.43 |
| Molecular function | GO:0004471 | malate dehydrogenase (decarboxylating) (NAD+) activity | 5 | 7 | 0.00204 | 71.43 |
| Molecular function | GO:0004573 | mannosyl-oligosaccharide glucosidase activity | 5 | 7 | 0.00204 | 71.43 |
| Molecular function | GO:0016615 | malate dehydrogenase activity | 5 | 7 | 0.00204 | 71.43 |
| Molecular function | GO:0004866 | endopeptidase inhibitor activity | 4 | 5 | 0.00339 | 80.00 |

| | | | | | | |
|---|---|---|---|---|---|---|
| Molecular function | GO:0004867 | serine-type endopeptidase inhibitor activity | 4 | 5 | 0.00339 | 80.00 |
| Molecular function | GO:0030414 | peptidase inhibitor activity | 4 | 5 | 0.00339 | 80.00 |
| Molecular function | GO:0061135 | endopeptidase regulator activity | 4 | 5 | 0.00339 | 80.00 |
| Molecular function | GO:0016705 | oxidoreductase activity, acting on paired donors, with incorporation or reduction of molecular oxygen | 38 | 151 | 0.00481 | 25.17 |
| Molecular function | GO:0016772 | transferase activity, transferring phosphorus-containing groups | 73 | 331 | 0.00555 | 22.05 |
| Molecular function | GO:0003678 | DNA helicase activity | 9 | 23 | 0.00874 | 39.13 |
| Molecular function | GO:0016491 | oxidoreductase activity | 119 | 593 | 0.01273 | 20.07 |
| Molecular function | GO:0005506 | iron ion binding | 33 | 136 | 0.01449 | 24.26 |
| Molecular function | GO:0003879 | ATP phosphoribosyltransferase activity | 3 | 4 | 0.01641 | 75.00 |
| Molecular function | GO:0004096 | catalase activity | 3 | 4 | 0.01641 | 75.00 |

| | | | | | | |
|---|---|---|---|---|---|---|
| Molecular function | GO:0004523 | RNA-DNA hybrid ribonuclease activity | 3 | 4 | 0.01641 | 75.00 |
| Molecular function | GO:0004748 | ribonucleoside-diphosphate reductase activity, thioredoxin disulfide as acceptor | 3 | 4 | 0.01641 | 75.00 |
| Molecular function | GO:0016725 | oxidoreductase activity, acting on CH or CH2 groups | 3 | 4 | 0.01641 | 75.00 |
| Molecular function | GO:0016728 | oxidoreductase activity, acting on CH or CH2 groups, disulfide as acceptor | 3 | 4 | 0.01641 | 75.00 |
| Molecular function | GO:0016872 | intramolecular lyase activity | 3 | 4 | 0.01641 | 75.00 |
| Molecular function | GO:0061731 | ribonucleoside-diphosphate reductase activity | 3 | 4 | 0.01641 | 75.00 |
| Molecular function | GO:0046914 | transition metal ion binding | 77 | 369 | 0.01796 | 20.87 |
| Molecular function | GO:0005524 | ATP binding | 108 | 540 | 0.01931 | 20.00 |
| Molecular function | GO:0032559 | adenyl ribonucleotide binding | 108 | 540 | 0.01931 | 20.00 |

| | | | | | | |
|---|---|---|---|---|---|---|
| Molecular function | GO:0030554 | adenyl nucleotide binding | 108 | 541 | 0.02034 | 19.96 |
| Molecular function | GO:0004553 | hydrolase activity, hydrolyzing O-glycosyl compounds | 35 | 153 | 0.02872 | 22.88 |
| Molecular function | GO:0004857 | enzyme inhibitor activity | 4 | 8 | 0.0311 | 50.00 |
| Molecular function | GO:0004932 | mating-type factor pheromone receptor activity | 4 | 8 | 0.0311 | 50.00 |
| Molecular function | GO:0016503 | pheromone receptor activity | 4 | 8 | 0.0311 | 50.00 |
| Molecular function | GO:0004934 | mating-type alpha-factor pheromone receptor activity | 3 | 5 | 0.03593 | 60.00 |
| Molecular function | GO:0036318 | peptide pheromone receptor activity | 3 | 5 | 0.03593 | 60.00 |
| Molecular function | GO:0004601 | peroxidase activity | 8 | 24 | 0.03606 | 33.33 |
| Molecular function | GO:0004497 | monooxygenase activity | 14 | 52 | 0.04271 | 26.92 |
| Molecular function | GO:0016798 | hydrolase activity, acting on glycosyl bonds | 35 | 159 | 0.04801 | 22.01 |
| Molecular function | GO:0001653 | peptide receptor activity | 4 | 9 | 0.0487 | 44.44 |

| | | | | | | |
|---|---|---|---|---|---|---|
| Molecular function | GO:0008528 | G-protein coupled peptide receptor activity | 4 | 9 | 0.0487 | 44.44 |
| Biological process | GO:0015074 | DNA integration | 23 | 25 | 1.7E-16 | 92.00 |
| Biological process | GO:0006259 | DNA metabolic process | 56 | 180 | 5.1E-07 | 31.11 |
| Biological process | GO:0008152 | metabolic process | 435 | 2374 | 4.5E-06 | 18.32 |
| Biological process | GO:0006468 | protein phosphorylation | 53 | 205 | 0.00033 | 25.85 |
| Biological process | GO:0000723 | telomere maintenance | 8 | 15 | 0.00119 | 53.33 |
| Biological process | GO:0032200 | telomere organization | 8 | 15 | 0.00119 | 53.33 |
| Biological process | GO:0060249 | anatomical structure homeostasis | 8 | 15 | 0.00119 | 53.33 |
| Biological process | GO:0009405 | pathogenesis | 4 | 5 | 0.00326 | 80.00 |
| Biological process | GO:0055114 | oxidation-reduction process | 116 | 562 | 0.0033 | 20.64 |
| Biological process | GO:0051276 | chromosome organization | 26 | 93 | 0.00363 | 27.96 |
| Biological process | GO:0043170 | macromolecule metabolic process | 246 | 1311 | 0.00376 | 18.76 |
| Biological process | GO:0044238 | primary metabolic process | 318 | 1745 | 0.00408 | 18.22 |

| | | | | | | |
|---|---|---|---|---|---|---|
| Biological process | GO:0016310 | phosphorylation | 54 | 233 | 0.00445 | 23.18 |
| Biological process | GO:0009403 | toxin biosynthetic process | 5 | 8 | 0.00447 | 62.50 |
| Biological process | GO:0009404 | toxin metabolic process | 5 | 8 | 0.00447 | 62.50 |
| Biological process | GO:0043385 | mycotoxin metabolic process | 5 | 8 | 0.00447 | 62.50 |
| Biological process | GO:0043386 | mycotoxin biosynthetic process | 5 | 8 | 0.00447 | 62.50 |
| Biological process | GO:0044710 | single-organism metabolic process | 200 | 1060 | 0.00928 | 18.87 |
| Biological process | GO:0071704 | organic substance metabolic process | 325 | 1816 | 0.01387 | 17.90 |
| Biological process | GO:0019748 | secondary metabolic process | 5 | 10 | 0.01505 | 50.00 |
| Biological process | GO:0044550 | secondary metabolite biosynthetic process | 5 | 10 | 0.01505 | 50.00 |
| Biological process | GO:0051704 | multi-organism process | 5 | 10 | 0.01505 | 50.00 |
| Biological process | GO:0043484 | regulation of RNA splicing | 3 | 4 | 0.01595 | 75.00 |
| Biological process | GO:0046165 | alcohol biosynthetic process | 4 | 7 | 0.01727 | 57.14 |

| | | | | | | |
|---|---|---|---|---|---|---|
| Biological process | GO:0046700 | heterocycle catabolic process | 11 | 34 | 0.01758 | 32.35 |
| Biological process | GO:0044260 | cellular macromolecule metabolic process | 217 | 1175 | 0.01793 | 18.47 |
| Biological process | GO:0019538 | protein metabolic process | 133 | 689 | 0.01925 | 19.30 |
| Biological process | GO:1901361 | organic cyclic compound catabolic process | 11 | 35 | 0.02192 | 31.43 |
| Biological process | GO:1901606 | alpha-amino acid catabolic process | 5 | 11 | 0.0239 | 45.45 |
| Biological process | GO:0006979 | response to oxidative stress | 7 | 19 | 0.02712 | 36.84 |
| Biological process | GO:0000105 | histidine biosynthetic process | 4 | 8 | 0.03006 | 50.00 |
| Biological process | GO:0006547 | histidine metabolic process | 4 | 8 | 0.03006 | 50.00 |
| Biological process | GO:0052803 | imidazole-containing compound metabolic process | 4 | 8 | 0.03006 | 50.00 |
| Biological process | GO:0009311 | oligosaccharide metabolic process | 5 | 12 | 0.0355 | 41.67 |
| Biological process | GO:0044270 | cellular nitrogen compound catabolic process | 10 | 33 | 0.03619 | 30.30 |

| | | | | | | | |
|---|---|---|---|---|---|---|---|
| | Biological process | GO:1901565 | organonitrogen compound catabolic process | 8 | 25 | 0.04316 | 32.00 |
| SP30 | Cellular component | GO:0000943 | retrotransposon nucleocapsid | 10 | 13 | 1.4E-07 | 76.92 |
| | Cellular component | GO:0045263 | proton-transporting ATP synthase complex, coupling factor F(o) | 7 | 13 | 0.00036 | 53.85 |
| | Cellular component | GO:0045259 | proton-transporting ATP synthase complex | 7 | 17 | 0.00264 | 41.18 |
| | Cellular component | GO:0033177 | proton-transporting two-sector ATPase complex, proton-transporting domain | 7 | 19 | 0.0055 | 36.84 |
| | Cellular component | GO:0005576 | extracellular region | 8 | 25 | 0.008 | 32.00 |
| | Cellular component | GO:0005618 | cell wall | 5 | 13 | 0.01551 | 38.46 |
| | Cellular component | GO:0009277 | fungal-type cell wall | 5 | 13 | 0.01551 | 38.46 |
| | Cellular component | GO:0030312 | external encapsulating structure | 5 | 13 | 0.01551 | 38.46 |
| | Cellular component | GO:0000118 | histone deacetylase complex | 3 | 5 | 0.01553 | 60.00 |
| | Cellular component | GO:0070824 | SHREC complex | 3 | 5 | 0.01553 | 60.00 |

| | | | | | | |
|---|---|---|---|---|---|---|
| Cellular component | GO:0017053 | transcriptional repressor complex | 3 | 6 | 0.02825 | 50.00 |
| Cellular component | GO:0090568 | nuclear transcriptional repressor complex | 3 | 6 | 0.02825 | 50.00 |
| Cellular component | GO:0016469 | proton-transporting two-sector ATPase complex | 7 | 27 | 0.04089 | 25.93 |
| Cellular component | GO:0005887 | integral component of plasma membrane | 2 | 3 | 0.04218 | 66.67 |
| Cellular component | GO:0031226 | intrinsic component of plasma membrane | 2 | 3 | 0.04218 | 66.67 |
| Cellular component | GO:0032592 | integral component of mitochondrial membrane | 2 | 3 | 0.04218 | 66.67 |
| Cellular component | GO:0098573 | intrinsic component of mitochondrial membrane | 2 | 3 | 0.04218 | 66.67 |
| Cellular component | GO:0032993 | protein-DNA complex | 5 | 17 | 0.04938 | 29.41 |
| Molecular function | GO:0003678 | DNA helicase activity | 23 | 40 | 2.4E-09 | 57.50 |
| Molecular function | GO:0004386 | helicase activity | 26 | 58 | 2E-07 | 44.83 |
| Molecular function | GO:0004523 | RNA-DNA hybrid ribonuclease activity | 6 | 7 | 0.0001 | 85.71 |

| | | | | | | |
|---|---|---|---|---|---|---|
| Molecular function | GO:0017111 | nucleoside-triphosphatase activity | 57 | 236 | 0.00066 | 24.15 |
| Molecular function | GO:0016462 | pyrophosphatase activity | 57 | 242 | 0.00128 | 23.55 |
| Molecular function | GO:0003777 | microtubule motor activity | 9 | 19 | 0.0014 | 47.37 |
| Molecular function | GO:0016818 | hydrolase activity, acting on acid anhydrides, in phosphorus-containing anhydrides | 57 | 245 | 0.00174 | 23.27 |
| Molecular function | GO:0016817 | hydrolase activity, acting on acid anhydrides | 57 | 248 | 0.00234 | 22.98 |
| Molecular function | GO:0016705 | oxidoreductase activity, acting on paired donors, with incorporation or reduction of molecular oxygen | 37 | 147 | 0.00273 | 25.17 |
| Molecular function | GO:0004866 | endopeptidase inhibitor activity | 4 | 5 | 0.00292 | 80.00 |
| Molecular function | GO:0004867 | serine-type endopeptidase inhibitor activity | 4 | 5 | 0.00292 | 80.00 |
| Molecular function | GO:0030414 | peptidase inhibitor activity | 4 | 5 | 0.00292 | 80.00 |

| | | | | | | |
|---|---|---|---|---|---|---|
| Molecular function | GO:0061135 | endopeptidase regulator activity | 4 | 5 | 0.00292 | 80.00 |
| Molecular function | GO:0020037 | heme binding | 36 | 143 | 0.00308 | 25.17 |
| Molecular function | GO:0046906 | tetrapyrrole binding | 36 | 143 | 0.00308 | 25.17 |
| Molecular function | GO:0016891 | endoribonuclease activity, producing 5'-phosphomonoesters | 7 | 15 | 0.00538 | 46.67 |
| Molecular function | GO:0004672 | protein kinase activity | 46 | 200 | 0.00595 | 23.00 |
| Molecular function | GO:0005506 | iron ion binding | 33 | 134 | 0.00648 | 24.63 |
| Molecular function | GO:0046914 | transition metal ion binding | 76 | 363 | 0.00675 | 20.94 |
| Molecular function | GO:0016893 | endonuclease activity, active with either ribo- or deoxyribonucleic acids and producing 5'-phosphomonoesters | 7 | 16 | 0.00826 | 43.75 |
| Molecular function | GO:0003774 | motor activity | 9 | 25 | 0.01248 | 36.00 |
| Molecular function | GO:0061134 | peptidase regulator activity | 4 | 7 | 0.01559 | 57.14 |

| | | | | | | |
|---|---|---|---|---|---|---|
| Molecular function | GO:0030246 | carbohydrate binding | 10 | 31 | 0.01963 | 32.26 |
| Molecular function | GO:0016301 | kinase activity | 50 | 236 | 0.02096 | 21.19 |
| Molecular function | GO:0001871 | pattern binding | 8 | 23 | 0.02259 | 34.78 |
| Molecular function | GO:0030247 | polysaccharide binding | 8 | 23 | 0.02259 | 34.78 |
| Molecular function | GO:0004521 | endoribonuclease activity | 7 | 19 | 0.02345 | 36.84 |
| Molecular function | GO:0016787 | hydrolase activity | 156 | 846 | 0.0237 | 18.44 |
| Molecular function | GO:0008521 | acetyl-CoA transporter activity | 2 | 2 | 0.02596 | 100.00 |
| Molecular function | GO:0022843 | voltage-gated cation channel activity | 2 | 2 | 0.02596 | 100.00 |
| Molecular function | GO:0051184 | cofactor transporter activity | 2 | 2 | 0.02596 | 100.00 |
| Molecular function | GO:0051185 | coenzyme transporter activity | 2 | 2 | 0.02596 | 100.00 |
| Molecular function | GO:0016773 | phosphotransferase activity, alcohol group as acceptor | 48 | 228 | 0.02607 | 21.05 |
| Molecular function | GO:0008017 | microtubule binding | 7 | 20 | 0.0312 | 35.00 |
| Molecular function | GO:0015926 | glucosidase activity | 5 | 12 | 0.0317 | 41.67 |

| | | | | | | |
|---|---|---|---|---|---|---|
| Molecular function | GO:0016772 | transferase activity, transferring phosphorus-containing groups | 64 | 320 | 0.03215 | 20.00 |
| Molecular function | GO:0004339 | glucan 1,4-alpha-glucosidase activity | 3 | 5 | 0.03234 | 60.00 |
| Molecular function | GO:2001070 | starch binding | 3 | 5 | 0.03234 | 60.00 |
| Molecular function | GO:0004857 | enzyme inhibitor activity | 4 | 9 | 0.04291 | 44.44 |
| Molecular function | GO:0005488 | binding | 414 | 2442 | 0.04417 | 16.95 |
| Molecular function | GO:0032559 | adenyl ribonucleotide binding | 99 | 527 | 0.04485 | 18.79 |
| Molecular function | GO:0005199 | structural constituent of cell wall | 5 | 13 | 0.04485 | 38.46 |
| Molecular function | GO:0030554 | adenyl nucleotide binding | 99 | 528 | 0.04687 | 18.75 |
| Biological process | GO:0015074 | DNA integration | 24 | 25 | 8.1E-19 | 96.00 |
| Biological process | GO:0000723 | telomere maintenance | 24 | 33 | 3.5E-13 | 72.73 |
| Biological process | GO:0032200 | telomere organization | 24 | 33 | 3.5E-13 | 72.73 |

| | | | | | | |
|---|---|---|---|---|---|---|
| Biological process | GO:0060249 | anatomical structure homeostasis | 24 | 33 | 3.5E-13 | 72.73 |
| Biological process | GO:0006259 | DNA metabolic process | 67 | 189 | 6.2E-12 | 35.45 |
| Biological process | GO:0042592 | homeostatic process | 28 | 65 | 1.2E-07 | 43.08 |
| Biological process | GO:0065008 | regulation of biological quality | 31 | 84 | 1.5E-06 | 36.90 |
| Biological process | GO:0051276 | chromosome organization | 35 | 103 | 3E-06 | 33.98 |
| Biological process | GO:0008152 | metabolic process | 403 | 2322 | 4.5E-05 | 17.36 |
| Biological process | GO:0006281 | DNA repair | 35 | 120 | 0.00012 | 29.17 |
| Biological process | GO:0006974 | cellular response to DNA damage stimulus | 35 | 125 | 0.0003 | 28.00 |
| Biological process | GO:0043170 | macromolecule metabolic process | 236 | 1282 | 0.00065 | 18.41 |
| Biological process | GO:0006950 | response to stress | 38 | 147 | 0.00093 | 25.85 |
| Biological process | GO:0033554 | cellular response to stress | 35 | 133 | 0.00104 | 26.32 |
| Biological process | GO:0006928 | movement of cell or subcellular component | 9 | 19 | 0.0012 | 47.37 |

| | | | | | | |
|---|---|---|---|---|---|---|
| Biological process | GO:0007018 | microtubule-based movement | 9 | 19 | 0.0012 | 47.37 |
| Biological process | GO:1902589 | single-organism organelle organization | 33 | 126 | 0.00158 | 26.19 |
| Biological process | GO:0006468 | protein phosphorylation | 47 | 198 | 0.00178 | 23.74 |
| Biological process | GO:0071704 | organic substance metabolic process | 312 | 1787 | 0.00237 | 17.46 |
| Biological process | GO:0044238 | primary metabolic process | 299 | 1710 | 0.0032 | 17.49 |
| Biological process | GO:0044260 | cellular macromolecule metabolic process | 209 | 1154 | 0.00465 | 18.11 |
| Biological process | GO:0015988 | energy coupled proton transmembrane transport, against electrochemical gradient | 7 | 16 | 0.00739 | 43.75 |
| Biological process | GO:0015991 | ATP hydrolysis coupled proton transport | 7 | 16 | 0.00739 | 43.75 |
| Biological process | GO:0090662 | ATP hydrolysis coupled transmembrane transport | 7 | 16 | 0.00739 | 43.75 |
| Biological process | GO:0006754 | ATP biosynthetic process | 8 | 20 | 0.00802 | 40.00 |

| | | | | | | |
|---|---|---|---|---|---|---|
| Biological process | GO:0015985 | energy coupled proton transport, down electrochemical gradient | 8 | 20 | 0.00802 | 40.00 |
| Biological process | GO:0015986 | ATP synthesis coupled proton transport | 8 | 20 | 0.00802 | 40.00 |
| Biological process | GO:0006996 | organelle organization | 42 | 188 | 0.0097 | 22.34 |
| Biological process | GO:0009142 | nucleoside triphosphate biosynthetic process | 8 | 21 | 0.0112 | 38.10 |
| Biological process | GO:0009145 | purine nucleoside triphosphate biosynthetic process | 8 | 21 | 0.0112 | 38.10 |
| Biological process | GO:0009201 | ribonucleoside triphosphate biosynthetic process | 8 | 21 | 0.0112 | 38.10 |
| Biological process | GO:0009206 | purine ribonucleoside triphosphate biosynthetic process | 8 | 21 | 0.0112 | 38.10 |
| Biological process | GO:0016310 | phosphorylation | 48 | 223 | 0.01207 | 21.52 |
| Biological process | GO:0007017 | microtubule-based process | 11 | 34 | 0.01241 | 32.35 |

| | | | | | | |
|---|---|---|---|---|---|---|
| Biological process | GO:0009163 | nucleoside biosynthetic process | 9 | 26 | 0.0145 | 34.62 |
| Biological process | GO:0042451 | purine nucleoside biosynthetic process | 9 | 26 | 0.0145 | 34.62 |
| Biological process | GO:0042455 | ribonucleoside biosynthetic process | 9 | 26 | 0.0145 | 34.62 |
| Biological process | GO:0046129 | purine ribonucleoside biosynthetic process | 9 | 26 | 0.0145 | 34.62 |
| Biological process | GO:1901659 | glycosyl compound biosynthetic process | 9 | 26 | 0.0145 | 34.62 |
| Biological process | GO:0006793 | phosphorus metabolic process | 74 | 373 | 0.01605 | 19.84 |
| Biological process | GO:0006796 | phosphate-containing compound metabolic process | 73 | 369 | 0.01788 | 19.78 |
| Biological process | GO:0090304 | nucleic acid metabolic process | 120 | 645 | 0.01864 | 18.60 |
| Biological process | GO:0050896 | response to stimulus | 56 | 276 | 0.02291 | 20.29 |
| Biological process | GO:0006450 | regulation of translational fidelity | 2 | 2 | 0.02499 | 100.00 |

| | | | | | | |
|---|---|---|---|---|---|---|
| Biological process | GO:0009127 | purine nucleoside monophosphate biosynthetic process | 8 | 24 | 0.02624 | 33.33 |
| Biological process | GO:0009156 | ribonucleoside monophosphate biosynthetic process | 8 | 24 | 0.02624 | 33.33 |
| Biological process | GO:0009168 | purine ribonucleoside monophosphate biosynthetic process | 8 | 24 | 0.02624 | 33.33 |
| Biological process | GO:0051716 | cellular response to stimulus | 53 | 262 | 0.02801 | 20.23 |
| Biological process | GO:0015672 | monovalent inorganic cation transport | 12 | 43 | 0.03029 | 27.91 |
| Biological process | GO:0006139 | nucleobase-containing compound metabolic process | 132 | 729 | 0.03206 | 18.11 |
| Biological process | GO:0009124 | nucleoside monophosphate biosynthetic process | 8 | 25 | 0.03342 | 32.00 |
| Biological process | GO:0065007 | biological regulation | 81 | 427 | 0.03449 | 18.97 |
| Biological process | GO:0006725 | cellular aromatic compound metabolic process | 138 | 770 | 0.03885 | 17.92 |

| | | | | | | |
|---|---|---|---|---|---|---|
| Biological process | GO:0006818 | hydrogen transport | 10 | 35 | 0.03958 | 28.57 |
| Biological process | GO:0015992 | proton transport | 10 | 35 | 0.03958 | 28.57 |
| Biological process | GO:1902600 | hydrogen ion transmembrane transport | 10 | 35 | 0.03958 | 28.57 |
| Biological process | GO:1901360 | organic cyclic compound metabolic process | 140 | 783 | 0.0402 | 17.88 |
| Biological process | GO:0034641 | cellular nitrogen compound metabolic process | 161 | 911 | 0.04022 | 17.67 |
| Biological process | GO:0051704 | multi-organism process | 4 | 9 | 0.04026 | 44.44 |
| Biological process | GO:0009152 | purine ribonucleotide biosynthetic process | 8 | 26 | 0.04183 | 30.77 |
| Biological process | GO:0009260 | ribonucleotide biosynthetic process | 8 | 26 | 0.04183 | 30.77 |
| Biological process | GO:0046390 | ribose phosphate biosynthetic process | 8 | 26 | 0.04183 | 30.77 |
| Biological process | GO:0072522 | purine-containing compound biosynthetic process | 9 | 31 | 0.04519 | 29.03 |

| | | | | | | |
|---|---|---|---|---|---|---|
| | Biological process | GO:0046483 | heterocycle metabolic process | 137 | 768 | 0.04553 | 17.84 |

## Table S10. KEGG term analysis of rearranged genes between SP3 and SP30

| Genome | KEGG_A_ class | KEGG_B_ class | Pathway ID | Pathway | out (319) | All (2086) | P value | K_IDs |
|---|---|---|---|---|---|---|---|---|
| SP3 | Organismal Systems | Aging | ko04213 | Longevity regulating pathway - multiple species | 15 | 34 | 4.94E-05 | K04345+K03283+K03283+K03283+K03781+K03283+K06067+K04564+K03781+K03695+K03781+K12767+K07203+K07203+K07827 |
| | Metabolism | Nucleotide metabolism | ko00240 | Pyrimidine metabolism | 15 | 40 | 0.0004433 | K10808+K10807+K10807+K10808+K01591+K10807+K10808+K06966+K06966+K10808+K10807+K10807+K10808+K03783+K13800 |
| | Metabolism | Metabolism of other amino acids | ko00480 | Glutathione metabolism | 16 | 46 | 0.0007621 | K10808+K10807+K10807+K10808+K00799+K00799+K00799+K07160+K10807+K10808+K01469+K18592+K10808+K10807+K10807+K10808 |
| | Genetic Information Processing | Transcription | ko03040 | Spliceosome | 25 | 103 | 0.009632 | K12858+K03283+K03283+K12812+K12832+K11096+K12840+K03283+K12856+K12843+K12840+K03283+K12856+K12860+K12860+K12867+K12840+K12870+K12840+K12834+K12815+K12815+K12815+K12812+K12844 |
| | Metabolism | Nucleotide metabolism | ko00230 | Purine metabolism | 16 | 62 | 0.0202741 | K10808+K10807+K10807+K10808+K00365+K01477+K10807+K10808+K06966+K06966+K10808+K10807+K10807+K01487+K10808+K03783 |
| | Genetic Information Processing | Folding, sorting and degradation | ko04141 | Protein processing in endoplasmic reticulum | 20 | 88 | 0.038509 | K03283+K09490+K12670+K09490+K03283+K03283+K01230+K06689+K03283+K01230+K13993+K13993+K09518+K13993+K10575+K10088+K14005+K13993+K10661+K09503 |
| | Metabolism | Metabolism of other amino acids | ko00460 | Cyanoamino acid metabolism | 7 | 23 | 0.0497053 | K05349+K05349+K01188+K05349+K05349+K18592+K13051 |
| | Metabolism | Carbohydrate metabolism | ko00053 | Ascorbate and aldarate metabolism | 5 | 15 | 0.0654789 | K00012+K00128+K01053+K00128+K00128 |
| | Environmental Information Processing | Signal transduction | ko04011 | MAPK signaling pathway - yeast | 18 | 85 | 0.086753 | K07973+K11246+K11229+K19860+K04627+K04627+K04627+K19860+K03781+K11227+K03114+K03781+K02220+K03781+K04627+K04563+K04371+K10591 |
| | Genetic Information Processing | Replication and repair | ko03430 | Mismatch repair | 8 | 33 | 0.1187127 | K10756+K03657+K10756+K10756+K10858+K10756+K10756+K10755 |

| | | | | | | | | |
|---|---|---|---|---|---|---|---|---|
| | Metabolism | Amino acid metabolism | ko00340 | Histidine metabolism | 7 | 29 | 0.1421542 | K00128+K00765+K00765+K01814+K00128+K00765+K00128 |
| | Cellular Processes | Cell growth and death | ko04113 | Meiosis - yeast | 14 | 68 | 0.1445152 | K04345+K03351+K03348+K02540+K03114+K02220+K12767+K07203+K07203+K02216+K04563+K06269+K02178+K07827 |
| | Metabolism | Carbohydrate metabolism | ko00040 | Pentose and glucuronate interconversions | 6 | 25 | 0.1712253 | K01213+K00012+K00963+K00008+K01213+K00008 |
| | Metabolism | Global and overview maps | ko01200 | Carbon metabolism | 20 | 106 | 0.1795809 | K14454+K01624+K15634+K00027+K00122+K00237+K01053+K17066+K03781+K00237+K03781+K00234+K03781+K00029+K00029+K00234+K00029+K00029+K00927+K17066 |
| | Metabolism | Metabolism of cofactors and vitamins | ko00790 | Folate biosynthesis | 4 | 15 | 0.1858025 | K03635+K20457+K20457+K01113 |
| | Metabolism | Energy metabolism | ko00910 | Nitrogen metabolism | 4 | 15 | 0.1858025 | K00459+K15371+K00459+K00459 |
| | Metabolism | Glycan biosynthesis and metabolism | ko00513 | Various types of N-glycan biosynthesis | 6 | 26 | 0.1958388 | K12670+K01230+K03847+K01230+K03847+K03847 |
| | Genetic Information Processing | Folding, sorting and degradation | ko04120 | Ubiquitin mediated proteolysis | 13 | 66 | 0.1980409 | K03361+K03361+K10577+K10686+K03351+K06689+K03348+K03178+K03361+K10575+K10590+K10591+K10582 |
| | Metabolism | Amino acid metabolism | ko00380 | Tryptophan metabolism | 9 | 43 | 0.2003667 | K00128+K01426+K03781+K03781+K03781+K00128+K00486+K01556+K00128 |
| | Genetic Information Processing | Translation | ko03015 | mRNA surveillance pathway | 12 | 62 | 0.2290515 | K03265+K12812+K15542+K03267+K03267+K03267+K03267+K03267+K03267+K06269+K12812+K00565 |
| SP30 | Environmental Information Processing | Signal transduction | ko04011 | MAPK signaling pathway - yeast | 26 | 91 | 0.0001741 | K07973+K10591+K19860+K19860+K04371+K04627+K04627+K04627+K02677+K02677+K02677+K19704+K19704+K11227+K03114+K12412+K02220+K06630+K04371+K02516+K19860+K19806+K00889+K19838+K09265+K09051 |
| | Genetic Information Processing | Transcription | ko03040 | Spliceosome | 24 | 102 | 0.0058575 | K12871+K12858+K12813+K12813+K12624+K12811+K12811+K12852+K12813+K12854+K12870+K12860+K12860+K12860+K12812+K12877+K12897+K12854+K12854+K12854+K12854+K12854+K12820+K12811 |

| | | | | | | | |
|---|---|---|---|---|---|---|---|
| Metabolism | Carbohydrate metabolism | ko00500 | Starch and sucrose metabolism | 14 | 54 | 0.0141855 | K01176+K05349+K05349+K01196+K01178+K01196+K01178+K01178+K00963+K05349+K05349+K01179+K01188+K01194 |
| Metabolism | Lipid metabolism | ko01040 | Biosynthesis of unsaturated fatty acids | 7 | 21 | 0.0202875 | K10256+K10256+K10256+K07513+K10256+K10256+K10256 |
| Genetic Information Processing | Folding, sorting and degradation | ko04120 | Ubiquitin mediated proteolysis | 16 | 68 | 0.0228466 | K03361+K03361+K10686+K10686+K10591+K10590+K03361+K10592+K10592+K04554+K03361+K03361+K10575+K03357+K10579+K03360 |
| Environmental Information Processing | Signal transduction | ko04070 | Phosphatidylinositol signaling system | 8 | 27 | 0.0275011 | K02677+K02677+K02677+K07756+K01099+K00889+K05857+K00915 |
| Metabolism | Global and overview maps | ko01212 | Fatty acid metabolism | 10 | 39 | 0.0384956 | K10256+K10256+K01897+K00249+K10256+K07513+K01897+K10256+K10256+K10256 |
| Environmental Information Processing | Membrane transport | ko02010 | ABC transporters | 4 | 10 | 0.0402952 | K05658+K05658+K05658+K05658 |
| Genetic Information Processing | Replication and repair | ko03440 | Homologous recombination | 7 | 25 | 0.0510998 | K10879+K10901+K10901+K10901+K10901+K10901+K10873 |
| Genetic Information Processing | Translation | ko03013 | RNA transport | 23 | 117 | 0.0538224 | K03257+K14288+K12160+K14290+K14290+K03260+K14315+K14288+K03259+K03259+K14325+K14309+K14288+K14298+K14298+K02516+K14326+K12812+K12877+K14303+K13126+K00784+K14288 |
| Metabolism | Metabolism of other amino acids | ko00460 | Cyanoamino acid metabolism | 6 | 22 | 0.0771401 | K05349+K05349+K05349+K05349+K18592+K01188 |
| Cellular Processes | Cell growth and death | ko04111 | Cell cycle - yeast | 17 | 92 | 0.1390532 | K03361+K03361+K02365+K06682+K04371+K06636+K02179+K03114+K12412+K02220+K03361+K04371+K03361+K03361+K02516+K03357+K03360 |
| Metabolism | Biosynthesis of other secondary metabolites | ko00232 | Caffeine metabolism | 1 | 1 | 0.1407767 | K00365 |
| Metabolism | Lipid metabolism | ko00561 | Glycerolipid metabolism | 7 | 32 | 0.1527004 | K00128+K00128+K15728+K15728+K07407+K07407+K13519 |

| | | | | | | | |
|---|---|---|---|---|---|---|---|
| Metabolism | Energy metabolism | ko00190 | Oxidative phosphorylation | 14 | 75 | 0.159289 | K03946+K02132+K02267+K03935+K02259+K02259+K02128+K02133+K02128+K02128+K02128+K02128+K02128+K02128 |
| Metabolism | Lipid metabolism | ko00071 | Fatty acid degradation | 7 | 33 | 0.1719216 | K00128+K00128+K01897+K00249+K07513+K01897+K00121 |
| Metabolism | Energy metabolism | ko00680 | Methane metabolism | 5 | 22 | 0.1876284 | K00122+K01624+K17066+K17066+K00121 |
| Metabolism | Metabolism of cofactors and vitamins | ko00860 | Porphyrin and chlorophyll metabolism | 4 | 18 | 0.2407169 | K21480+K01719+K02259+K02259 |
| Metabolism | Lipid metabolism | ko00600 | Sphingolipid metabolism | 5 | 25 | 0.2698795 | K12351+K07407+K07407+K04708+K04709 |
| Metabolism | Glycan biosynthesis and metabolism | ko00603 | Glycosphingolipid biosynthesis - globo and isoglobo series | 2 | 8 | 0.313555 | K07407+K07407 |

**Table S11. Unique genes and primers for PCR analysis**

| No | Gene name | Chromosome | Length (bp) | Function | Primer name | Primer sequence |
|---|---|---|---|---|---|---|
| 1 | gene-Led00368-sp3 | Superscaffold1 | 1197 | hypothetical protein | 00368.1-F | TACCTTCCTCTCCCACTCGG |
| | | | | | 00368.1-R | AATCTGCCTTTCCTCAGCCC |
| 2 | gene-Led02602-sp3 | Superscaffold2 | 2241 | ribonucleoside-diphosphate reductase subunit M1 | 02602.1-F | CATGGAGCGCTTGAAAGTCG |
| | | | | | 02602.1-R | CTTCGCGATCATCAGCTTGC |
| 3 | gene-Led04647-sp3 | Superscaffold3 | 2546 | ATP-dependent DNA helicase PIF1 | 04647.1-F | CTCTCATGCCGACCGCTATT |
| | | | | | 04647.1-R | GTCCTCTGCGGTGACTTTCA |
| 4 | gene-Led05113-sp3 | Superscaffold4 | 1414 | hypothetical protein | 05113.1-F | CGGCGTGTTGGAACATGATC |
| | | | | | 05113.1-R | CACCAGGGCTGAAATGCAAC |
| 5 | gene-Led06191-sp3 | Superscaffold5 | 688 | alternative cyclin pcl1 | 06191.1-F | AACATCGAGTGCAGCCTTCA |
| | | | | | 06191.1-R | AAAGCACCGCGTTACACATG |
| 6 | gene-Led08788-sp3 | Superscaffold7 | 688 | hypothetical protein | 08788.1-F | AAAGCACCGCGTTACACATG |
| | | | | | 08788.1-R | AATGCTTGGAGTAGCTGCGT |
| 7 | gene-Led09905-sp3 | Superscaffold8 | 685 | alternative cyclin pcl1 | 09905.1-F | GTCCCGGTTGACACATTCCT |
| | | | | | 09905.1-R | AACATCGAGTGCAGCCTTGA |
| 8 | gene-Led07817-sp3 | Superscaffold6 | 1992 | Holliday junction resolvase | 07817.1-F | ACACAGACGTTTCGGCTCAT |
| | | | | | 07817.1-R | TGCCATCGACGTGAATGGAA |
| 9 | gene-Led10404-sp3 | Superscaffold9 | 638 | serine/threonine-protein kinase | 10404.1-F | CAGATAGGTGACGGTGGCTC |
| | | | | | 10404.1-R | CGGTGACATATGCCCTTGCT |
| 10 | gene-Led01615-sp3 | Superscaffold10 | 2035 | hypothetical protein | 01615.1-F | ATATTGCCAAACGCGCTGAC |
| | | | | | 01615.1-R | AGATGACCACCGCTGACAAG |
| 11 | gene-Led02286-sp30 | Superscaffold1 | 5723 | DNA helicase | 02286.1-F | GCAATCCGAAGCATTCCGTC |
| | | | | | 02286.1-R | CCGAATTCTTGGCCTCCTGT |
| 12 | gene-Led04228-sp30 | Superscaffold3 | 2546 | Glucose-methanol-choline oxidoreductase | 04228.1-F | AGTGAATTCCAGGTCCAGCG |
| | | | | | 04228.1-R | GGAGGTCAGTTCTCCTGTGC |

| | | | | | | |
|---|---|---|---|---|---|---|
| 13 | gene-Led05642-sp30 | Superscaffold4 | 2517 | serine threonine protein kinase [*Lentinula edode*s] | 05642.1-F | GCCCGTTCCAAACAAAGCAT |
| | | | | | 05642.1-R | AGAGTCTTGACACGCATCGG |
| 14 | gene-Led08536-sp30 | Superscaffold6 | 1524 | 8-amino-7-oxononanoate synthase | 08536.1-F | TCAAGGCCACTACACTGCTG |
| | | | | | 08536.1-R | ATCCCTCAGGCTGGAGATGT |
| 15 | gene-Led09638-sp30 | Superscaffold7 | 4290 | hypothetical protein | 09638.1-F | TCGCCCTTTGCCTTTCAGAT |
| | | | | | 09638.1-R | TCTGGCAAAACTGCGAATGC |
| 16 | gene-Led09779-sp30 | Superscaffold8 | 1964 | ribosomal protein L32 | 09779.1-F | CGAGTTCAACGACCCCAGAA |
| | | | | | 09779.1-R | TCCAGGAGCCCGAGGATAAA |
| 17 | gene-Led10568-sp30 | Superscaffold9 | 2547 | hypothetical protein LENED_003051 [*Lentinula edodes*] | 10568.1-F | ACATAGCAGTCGGTCCTCCT |
| | | | | | 10568.1-R | GGCTCGGTGACATCCATAGG |
| 18 | gene-Led02816-sp30 | Superscaffold10 | 1008 | hypothetical protein | 02816.1-F | TCAGGCTGAGGCATCTACCT |
| | | | | | 02816.1-R | GTCGGACAAACATGGGTTGC |

**Table S12. PCR and Sanger sequencing identity of the unique genes within each of two genomes**

| No | Gene name | DNA temple | Identity of PCR product |
|---|---|---|---|
| | | SP3 | 98% |
| 1 | gene-Led00368-sp3 | SP30 | 63% |
| | | 3×30 | 98% |
| | | SP3 | 98% |
| 2 | gene-Led02602-sp3 | SP30 | band with unexpected size |
| | | 3×30 | 98% |
| | | SP3 | 97% |
| 3 | gene-Led04647-sp3 | SP30 | no band |
| | | 3×30 | 97% |
| | | SP3 | 98% |
| 4 | gene-Led05113-sp3 | SP30 | no band |
| | | 3×30 | 98% |
| | | SP3 | 98% |
| 5 | gene-Led06191-sp3 | SP30 | no band |
| | | 3×30 | 98% |
| | | SP3 | 98% |
| 6 | gene-Led08788-sp3 | SP30 | no band |
| | | 3×30 | 98% |
| | | SP3 | 90% |
| 7 | gene-Led09905-sp3 | SP30 | no band |
| | | 3×30 | 90% |
| | | SP3 | 98% |
| 8 | gene-Led07817-sp3 | SP30 | no band |
| | | 3×30 | 98% |
| | | SP3 | 98% |
| 9 | gene-Led10404-sp3 | SP30 | no band |
| | | 3×30 | 98% |

| | | | |
|---|---|---|---|
| | | SP3 | 97% |
| 10 | gene-Led01615-sp3 | SP30 | band with unexpected size |
| | | 3×30 | 97% |
| | | SP3 | 15% |
| 11 | gene-Led02286-sp30 | SP30 | 97% |
| | | 3×30 | 97% |
| | | SP3 | no band |
| 12 | gene-Led04228-sp30 | SP30 | 98% |
| | | 3×30 | 98% |
| | | SP3 | no band |
| 13 | gene-Led05642-sp30 | SP30 | 99% |
| | | 3×30 | 99% |
| | | SP3 | band with unexpected size |
| 14 | gene-Led08536-sp30 | SP30 | 99% |
| | | 3×30 | 99% |
| | | SP3 | band with unexpected size |
| 15 | gene-Led09638-sp30 | SP30 | 99% |
| | | 3×30 | 99% |
| | | SP3 | band with unexpected size |
| 16 | gene-Led09779-sp30 | SP30 | 99% |
| | | 3×30 | 99% |
| | | SP3 | no band |
| 17 | gene-Led10568-sp30 | SP30 | 99% |
| | | 3×30 | 99% |
| | | SP3 | band with unexpected size |
| 18 | gene-Led02816-sp30 | SP30 | 99% |
| | | 3×30 | 99% |

**Table S13. The numbers of retrotransposons belonging to various long terminal repeat (LTR) superfamilies in the two monokaryotic strains**

| LTR superfamily | **SP3** | **SP30** |
|---|---|---|
| **Gypsy** | 12681 | 11170 |
| **Copia** | 1526 | 1326 |
| **Ngaro** | 76 | 227 |
| **Unknown** | 1633 | 1303 |
| **Total** | 15916 | 14026 |
